# Characteristics of *Staphylococcus saprophyticus* Isolated from Humans and Animals

**DOI:** 10.3390/ijms26146885

**Published:** 2025-07-17

**Authors:** Paulina Prorok, Karolina Bierowiec, Milena Skrok, Magdalena Karwańska, Magdalena Siedlecka, Marta Miszczak, Marta Książczyk, Katarzyna Kapczyńska, Krzysztof Rypuła

**Affiliations:** 1 Department of Epizootiology and Clinic of Birds and Exotic Animals, Division of Infectious Diseases and Veterinary Sciences, 50-365 Wrocław, Poland; milena.skrok@upwr.edu.pl (M.S.); magdalena.karwanska@upwr.edu.pl (M.K.); magdalena.siedlecka@upwr.edu.pl (M.S.); marta.miszczak@upwr.edu.pl (M.M.); krzysztof.rypula@upwr.edu.pl (K.R.); 2 Department of Microbiology, Faculty of Biological Sciences, University of Wroclaw, 51-148 Wrocław, Poland; marta.ksiazczyk@uwr.edu.pl; 3 Laboratory of Medical Microbiology, Hirszfeld Institute of Immunology and Experimental Therapy, Polish Academy of Sciences, 53-114 Wrocław, Poland; katarzyna.kapczynska@hirszfeld.pl

**Keywords:** epidemiology, public health, multidrug resistance, staphylococcus, CoNS

## Abstract

*Staphylococcus saprophyticus* (*S*. *saprophyticus*) is an opportunistic coagulase-negative staphylococcus (CoNS) known to cause urinary tract infections in humans and is increasingly recognized in veterinary medicine. The aim of this study was to provide an epidemiological characterization of *S*. *saprophyticus* strains and to identify potential virulence factors that may contribute to interspecies transmission. This research is particularly important, as companion animals represent an understudied reservoir of this microorganism, and their role in the spread of resistant pathogens remains insufficiently understood. A total of 61 *S*. *saprophyticus* strains isolated from humans, dogs, and cats were analyzed. Identification was performed using MALDI-TOF MS and confirmed by PCR targeting the *hrcA* gene. Antimicrobial susceptibility was assessed using the disk diffusion and broth microdilution methods, while resistance genes were detected by PCR. The *blaZ* and *mecA* genes were present in all strains; additionally, the majority harbored the resistance genes *ermA*, *ermB*, *tetM*, and *tetK*. Multidrug resistance (MDR) was identified in 21/61 strains (34.4%). Biofilm-forming capacity was temperature-dependent, with the strongest biofilm production observed at 37 °C (70.5%). At 38 °C and 39 °C, the proportion of strong biofilm producers decreased to 50.8% and 52.5%, respectively. All tested strains demonstrated pathogenic potential in the *Galleria mellonella* larvae infection model, with the highest mortality recorded for selected feline and canine strains. These findings indicate that *S*. *saprophyticus* strains from both humans and companion animals possess notable virulence and multidrug resistance. The detection of genotypically and phenotypically resistant strains in animals highlights their potential role as reservoir for zoonotic transmission.

## 1. Introduction

The vast majority of *Staphylococcus* species are coagulase-negative staphylococci (CoNS) [1]. For a long time, the pathogenicity of CoNS was questioned, and their presence in clinical samples was often regarded as contamination rather than true infection [1,2,3]. Since the 1980s, increasing attention has been paid to the clinical relevance of CoNS, which are now recognized as potential causes of serious nosocomial and environmental infections [4]. Today, certain CoNS species demonstrate pathogenicity comparable to that of *Staphylococcus aureus* (*S*. *aureus*) [5].

*Staphylococcus saprophyticus* (*S*. *saprophyticus*) naturally colonizes the skin and gastrointestinal tract of both humans and animals, particularly the perianal region and the genitourinary tract [6,7,8]. It is responsible for 20–42% of all human genitourinary infections, especially among young women [9,10,11]. Although urinary tract infections (UTIs) caused by *S*. *saprophyticus* are typically benign [12], there are reports in the literature of more severe nosocomial complications, including cystitis, pyelonephritis, urethritis, and even bacteremia and endocarditis [13,14,15,16,17]. Experimental murine models have further shown that *S*. *saprophyticus* displays a tropism for the kidneys, persisting in renal tissue for up to 14 days post-infection, while being rapidly cleared from the bladder. This finding supports the potential for kidney-specific pathology in certain hosts [18].

In veterinary medicine, livestock such as cattle and pigs have been identified as potential sources of zoonotic infection in humans. In the case of *S*. *saprophyticus,* a significant proportion of livestock animals have been shown to carry CoNS. One study reported the presence of *S*. *saprophyticus* in 7.1% of samples collected from the rectum of cattle carcasses and in 7.3% of samples obtained from pigs. These findings highlight the zoonotic potential of this microorganism and emphasize the need for further investigation into its role in interspecies transmission [19]. Given the documented carriage of *S*. *saprophyticus* in slaughter animals, increasing attention has been directed to its potential transmission to humans via contaminated food products. Infection with *S*. *saprophyticus* via consumption of meat from colonized animals has been described in the literature as a likely route of transmission. In a study by Hedman et al. exploring potential sources of UTIs caused by *S*. *saprophyticus*, the bacterium was detected in 16.4% of tested meat samples. The highest prevalence was found in raw pork and beef, where *S*. *saprophyticus* was present in up to 34% of samples [20].

Information regarding colonization and infection caused by *S*. *saprophyticus* in companion animals remains limited. In companion animals such as dogs and cats presenting with symptoms of UTIs, *S*. *saprophyticus* is estimated to be responsible for approximately 2.9% of UTI cases in dogs [21]. Additionally, this bacterium has been identified in dogs diagnosed with dermatitis, suggesting a potential pathogenic role in dermatological conditions as well [22]. In veterinary medicine, cases of disease caused by *S*. *saprophyticus* infection secondary to bladder stones in a female dog have been described [23], and even a fatal course of infection has been reported in armadillos [24]. In a study analyzing isolates of CoNS and coagulase-positive staphylococci (CoPS) obtained from both healthy and sick dogs and cats, a significantly higher proportion of methicillin-resistant strains was observed among CoNS (17.86%) compared to CoPS (1.95%). These findings suggest that the clinical relevance of CoNS may be underestimated, particularly given their increasing antibiotic resistance and potential role in infections in companion animals [25].

CoNS have increasingly been recognized as a significant public health concern due to their growing antibiotic resistance. These strains, long considered non-pathogenic and often dismissed as contaminants in clinical samples, are now acknowledged as important opportunistic pathogens, particularly in hospital settings [3,26]. CoNS are frequently isolated from bloodstream infections, catheter-associated infections, and prosthetic-device-related complications. Their ability to form biofilm significantly hinders treatment efficacy [27,28]. Studies have shown that CoNS strains can develop high levels of antibiotic resistance, particularly to β-lactams (e.g., oxacillin), as well as macrolides, lincosamides, and glycopeptides [29,30]. In some clinical settings, the proportion of methicillin-resistant CoNS isolates has exceeded 80–90%, indicating strong selective pressure imposed by antimicrobial therapies [1]. Moreover, CoNS are considered important reservoirs of resistance genes, including *mecA*, which may be horizontally transferred to other species such as *S*. *aureus*, increasing the risk of MRSA emergence [31]. There is also a noted increase in multidrug-resistant isolates, not only in healthcare environments but also among healthy individuals in the general population, emphasizing the need to broaden epidemiological surveillance beyond clinical setting [32,33]. *S*. *saprophyticus,* a species frequently isolated from companion animals, has also demonstrated considerable resistance to selected antimicrobials. One study revealed strong resistance to novobiocin and nalidixic acid, as well as a bimodal susceptibility pattern to erythromycin [34,35]. Notably, CoNS were isolated from 81.75% of 274 animals tested (224/274), with *S*. *saprophyticus* being the most identified species in sick dogs. In the same cohort, 70 methicillin-resistant strains were found among 392 isolates (70/392) [25].

Despite the increasing recognition of *S*. *saprophyticus* as a relevant opportunistic pathogen, there remains a critical knowledge gap regarding whether strains isolated from companion animals differ systematically from human isolates in terms of their antimicrobial resistance profiles and biofilm-forming capacities. This gap hinders our ability to assess the zoonotic potential of *S*. *saprophyticus* and to evaluate whether companion animals may act as reservoirs of particularly resistant or virulent strains. Therefore, the present study was designed to test the hypothesis that *S*. *saprophyticus* strains of animal origin demonstrate significantly higher resistance rates to selected antimicrobials and a greater capacity for biofilm formation compared to strains isolated from humans. Such findings would support the notion that companion animals contribute to the dissemination of zoonotic, multidrug-resistant *S*. *saprophyticus* strains with enhanced pathogenicity.

The present study aimed to investigate whether significant differences exist between *S*. *saprophyticus* strains of human and animal origin in terms of their antimicrobial resistance patterns and biofilm-forming properties, thereby contributing to a better understanding of their epidemiological significance in veterinary medicine and potential zoonotic implications for public health.

## 2. Results

### 2.1. Identification and Cluster Analysis

All archived strains (n = 61) were re-identified as *S*. *saprophyticus* using matrix-assisted laser desorption/ionization time-of-flight mass spectrometry (MALDI-TOF MS), with identification scores exceeding the threshold value of 2.000, indicating high reliability. Additionally, species affiliation of all strains was confirmed by molecular analysis through the detection of the *hrcA* gene, which is specific to *S*. *saprophyticus* [36].

**Figure 1:**
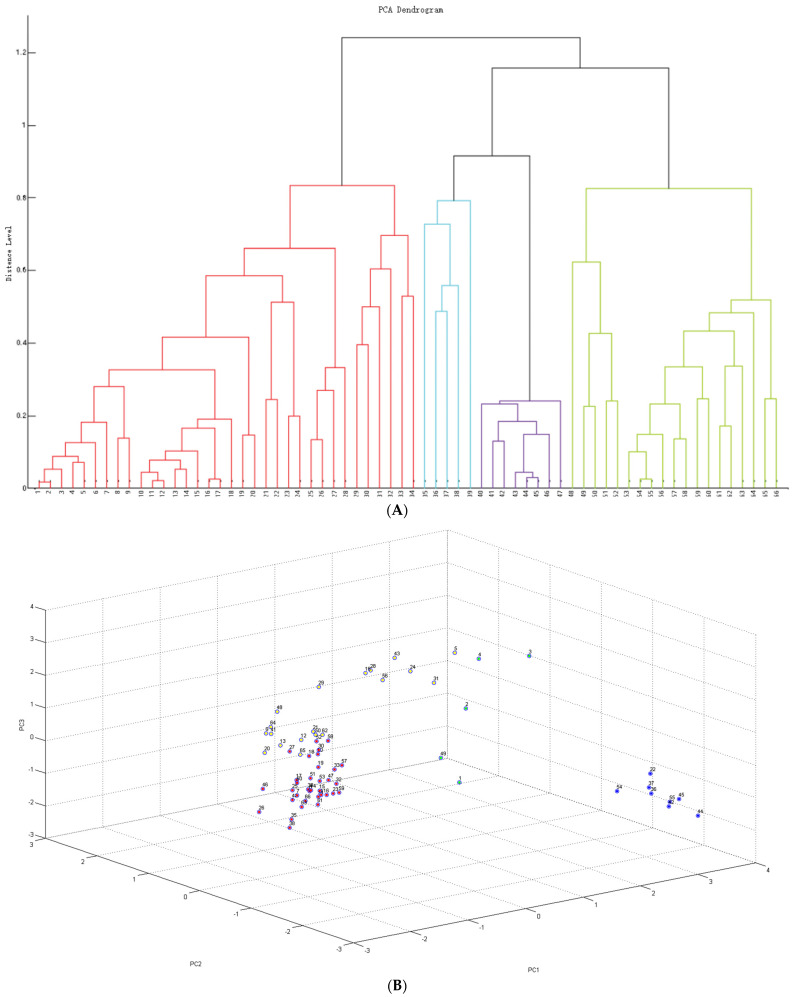
(**A**) Visual depiction of the closeness of individual spectra of *S*. *saprophyticus* and chosen referential strains obtained with MALDI-TOF MS analysis. (**B**) PCA clustering of MALDI Biotyper spectra of *S*. *saprophyticus* isolates and chosen referential strains. **1**. hHo8 **2**. hFe29 **3**. sHo2 **4**. hFe9 **5**. hHo12 **6**. hHo9 **7**. hFe26 **8**. hHo4 **9**. hHo5 **10**. sFe1 **11**. hFe2 **12**. hFe20 **13**. hFe6 **14**. sHo7 **15**. hFe3 **16**. hFe8 **17**. hFe27 **18**. hFe17 **19**. hHo1 **20**. hHo11 **21**. sCa7 **22**. hFe16 **23**. sCa8 (rejected) **24**. hFe11 **25**. hHo2 **26**. hFe19 **27**. sCa3 **28**. sCa4 **29**. hFe1 **30**. hFe25 **31**. hFe24 **32**. hHo14 **33**. sHo3 **34**. sHo5 **35**. *S*. *haemolyticus* PCM 2113 **36**. *S. aureus* ATCC 43300 **37**. *S*. *lugdunensis* PCM 2430 **38**. *S*. *epidermidis* PCM 2532 **39**. hHo12 **40**. hFe5 **41**. hFe10 **42**. hFe21 **43**. hFe13 **44**. hFe14 **45**. hFe15 **46**. hFe22 **47**. sFe3 **48**. *S. saprophyticus* PCM 2109 **49**. sCa2 **50**. hHo13 **51**. hFe7 **52**. sHo6 **53**. hHo10 **54**. sFe2 **55**. sCa5 **56**. hHo6 **57**. sHo4 **58**. hCa1 **59**. sCa1 **60**. hHo7 **61**. hHo3 **62**. hFe23 **63**. sHo1 **64**. hFe12 **65**. hFe4 **66**. hFe28.

To visualize the relationships among the analyzed strains, cluster analysis was performed using principal component analysis (PCA) and a hierarchical dendrogram (Figure 1A). Cluster analysis of MALDI-TOF MS spectra of strains identified as *S*. *saprophyticus* revealed the presence of four clearly defined clusters: red, blue, green, and purple (Figure 1A,B). The two main clusters (red and green) comprised isolates of various origins. Notably, human-derived strains—regardless of host species or health status—were distributed across different groups. The highest degree of homogeneity was observed among isolates obtained from healthy cats (hFe5, hFe10, hFe21, hFe13, hFe14, hFe15, hFe22), which clustered together within the purple group (Figure 1A,B). However, it is worth noting that most strains from healthy cats were assigned to other clusters. Notably, one feline strain (hFe12) deviated from the expected grouping and clustered together with the reference strains (*S*. *saprophyticus* PCM 2109, *S*. *aureus* ATCC 43300, *S*. *epidermidis* PCM 2532). Detailed results are shown in Figure 1A, which presents the principal component analysis (PCA) and the corresponding hierarchical clustering dendrogram.

### 2.2. Antimicrobial Phenotyping Testing

#### 2.2.1. Disk Diffusion Method

Antibiotic susceptibility of all *S*. *saprophyticus* strains (n = 61) was determined using the disk diffusion method, following current interpretive guidelines [37,38,39,40]. During the analysis, the zones of growth inhibition around antibiotic-impregnated disks were measured, allowing for the classification of the strains as susceptible, intermediate, or resistant. The analysis revealed that 34.43% (21/61) of the tested strains exhibited a multidrug resistance (MDR) phenotype, defined as resistance to at least one agent in three or more antimicrobial classes [41]. Among the human isolates, the most common resistance profiles were ampicillin (AMP), erythromycin (E), fusidic acid (FD) (n = 5) and ampicillin (AMP), amoxicillin with clavulanic acid (AMC), penicillin (P), and oxacillin (OX) (n = 2). In isolates obtained from other sources, a much greater diversity of resistance profiles was observed, with no repeating patterns. The complete phenotypic resistance profiles for all strains are provided in the Supplementary Material (Table S1).

In the detailed analysis, the highest overall resistance levels were observed for erythromycin (47.54%, 29/61), oxacillin (42.62%, 26/61), ampicillin (27.87%, 17/61), and fusidic acid (24.59%, 15/61). Conversely, the lowest resistance—levels or complete susceptibility—were recorded for mupirocin (3.28%, 2/61), tigecycline (1.64%, 1/61), linezolid (1.64%, 1/61), and trimethoprim with sulfamethoxazole (0/61). In the subgroup of strains obtained from humans (n = 21), the most prevalent resistance was noted against erythromycin (52.38%, 11/21) and oxacillin (42.86%, 9/21). No resistance was observed among human strains to linezolid, mupirocin, tigecycline, or trimethoprim with sulfamethoxazole.

Due to the limited sample size of some animal subgroups, the data from dogs and cats were analyzed together (n = 40). Similarly to the human strains results, high resistance to erythromycin (45.0%, 18/40) and oxacillin (42.5%, 17/40) was observed in the overall animal group, and no resistance was observed for tigecycline and sulfamethoxazole with trimethoprim. Among sick animals (n = 10), resistance rates were even higher, reaching 80.0% for both erythromycin and oxacillin. The lowest resistance levels overall were recorded in strains from clinically healthy animals, suggesting a potential correlation between host health status and antimicrobial resistance profiles. Detailed antibiotic susceptibility results are presented in Table 1.

#### 2.2.2. Minimal Inhibitory Concentration and Minimal Bactericidal Concentration for Selected Antimicrobials

In the analyzed group of strains (n = 61), no resistance was observed to vancomycin or teicoplanin. Erythromycin (78.69%, 48/61), clindamycin (44.26%, 27/61), and tetracycline (16.39%, 10/61) showed the highest overall resistance. Resistance among human isolates reached 95.24% (20/21) for erythromycin and 33.33% (7/21) for clindamycin. In comparison, strains derived from animals (n = 40) showed resistance levels of 70.0% (28/40) and 50.0% (20/40), respectively, with the highest rates recorded in strains from sick cats and dogs (100.0%, 10/10). Strains obtained from healthy animals showed lower resistance rates, particularly to tetracycline (13.33%, 4/30) and oxacillin (16.67%, 5/30), although the values remained relatively high. Minimum inhibitory concentrations (MICs) determined for selected antibiotics (vancomycin, teicoplanin, tetracycline, clindamycin, erythromycin, and oxacillin) were consistent with the results obtained using the disk diffusion method, exhibiting the same resistance trend.

**Figure 2:**
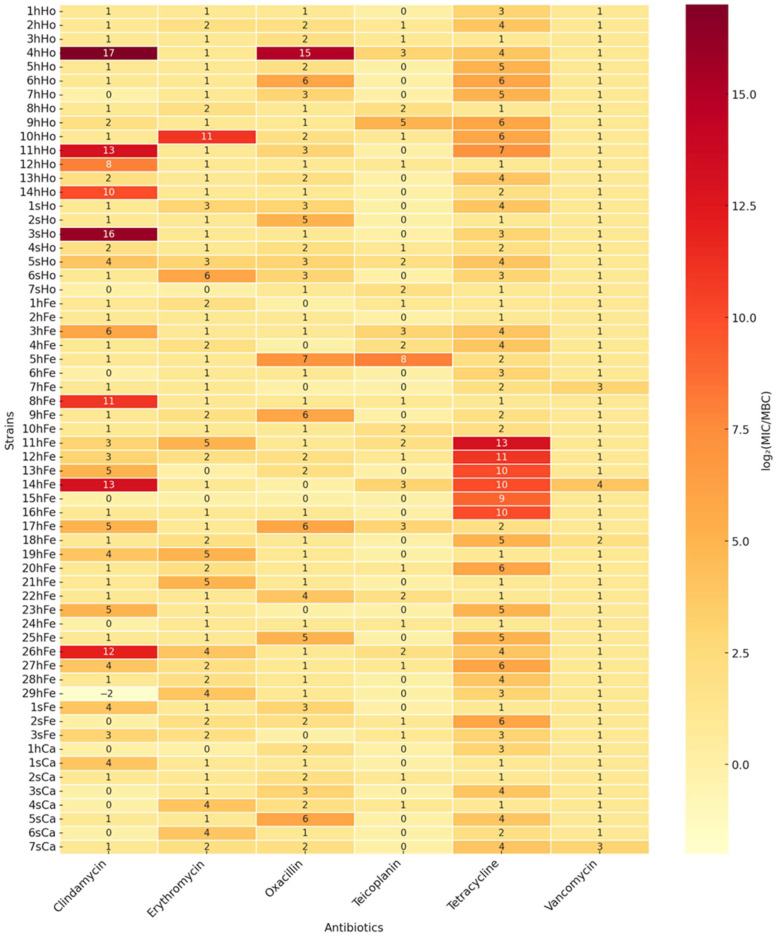
Heatmap illustrating the logarithmic ratio (log_2_) between the minimum bactericidal concentration (MBC) and the minimum inhibitory concentration (MIC) for 61 strains tested against six antibiotics: clindamycin, erythromycin, oxacillin, teicoplanin, tetracycline, and vancomycin. Darker colors indicate a greater difference between the MBC and MIC values (i.e., higher concentrations were required to achieve bactericidal effects). Lighter shades reflect lower MBC/MIC ratios, corresponding to stronger bacterial efficacy. Exact log_2_(MBC/MIC) values are numerically displayed on the heatmap.

This trend in resistance was also reflected in the bacterial activity profiling. To further assess the strength of antimicrobial activity, the relationship between the minimum bacterial concentration (MBC) and the minimum inhibitory concentration (MIC) was analyzed by calculating the log_2_(MBC/MIC) ratio (Figure 2). Detailed data are provided in Table S2 in the Supplementary Materials.

### 2.3. Detection of Resistance Genes Using PCR-Based Genotypic Analysis

The study revealed that the *blaZ* and *mecA* genes were detected in all analyzed strains. Macrolide resistance genes (*ermA*, *ermB*) were most frequently detected in human isolates, regardless of clinical status. Both genes were found in 92.86% (13/14) of strains from healthy individuals. In sick individuals, *ermA* was present in 85.71% (6/7), while *ermB* was found in 57.14% (4/7). In animal isolates, the prevalence of these genes was also high, though slightly lower—87.5% (35/40) for *ermA*, and 60.0% (24/40) for *ermB*. Among the four tetracycline resistance genes analyzed (*tetK*, *tetL*, *tetM*, *tetO*), *tetK* was not detected in any of the tested samples. In contrast, the remaining three genes were present, with *tetM* and *tetL* being the most prevalent, followed by *tetO*, which was detected less frequently. Regardless of clinical status, *tetM* was found in 100.0% of the tested strains from humans (21/21) and dogs (8/8). A similar trend was observed in cats, with a high prevalence of 96.88% (31/32). The *tetL* gene was detected in all diseased human isolates and was also common in the overall human group, with a frequency of 80.95% (17/21). The *fusB* gene was detected sporadically—13.79% (4/29)—and was detected exclusively in healthy cats. In these cases, the results of the disk diffusion test for fusidic acid resistance were consistent with the presence of the genes. Interestingly, the highest level of phenotypic resistance to fusidic acid, as determined by disk diffusion, was observed among diseased humans, despite the absence of any known resistance genes associated with this antibiotic. In the group of healthy cats, the *mupA* gene was identified in 6.90% (2/29) of individuals, and resistance to mupirocin, determined by the disk diffusion method, was consistent with the genotypic finding. Overall, resistance genes were more frequently detected in human isolates than in those from animals. A detailed breakdown of the genotypic antibiotic resistance data is presented in Table 2. Genetic profiles of resistance are shown in Table S4 in the Supplementary Materials.

### 2.4. Analysis of Bacterial Growth Curves and Biofilm Formation

Growth curves were generated for eleven strains (hHo4, hHo5, sHo1, sHo2, hFe14, hFe17, hFe26, sFe3, hCa1, sCa1, and sCa7) at temperatures of 37 °C, 38 °C, and 39 °C. The resulting growth curves, along with the corresponding doubling times, are presented in the Supplementary Materials (Figures S1–S10). Incubation at 37 °C promoted the most robust growth in the majority of the tested strains, regardless of host origin and health status. At this temperature, the strains exhibited the fastest growth rates and reached the highest OD_600_ plateau values, indicating that 37 °C represents optimal culture conditions for this species. At 38 °C, although the exponential growth rate remained similar to that at 37 °C, the OD_600_ plateau values were generally lower. In contrast, growth at 39 °C was clearly reduced for some strains—particularly those derived from healthy hosts. These strains displayed a prolonged lag phase and lower OD_600_ values after 24 h.

**Figure 3:**
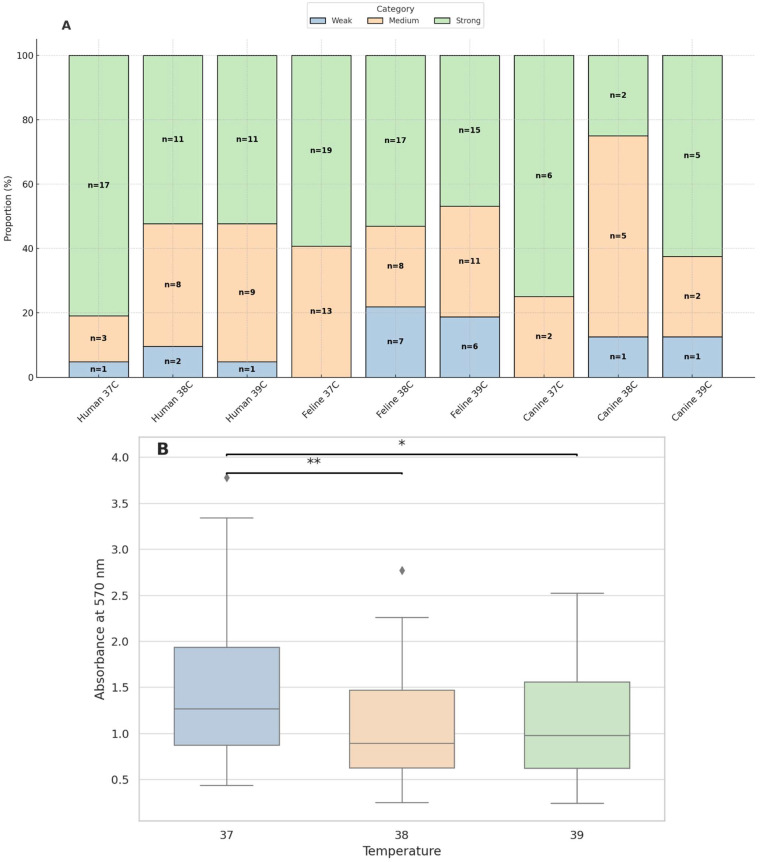
(**A**) Distribution of *S*. *saprophyticus* isolates by biofilm-forming strength (weak, moderate, strong) across different host origins (human, feline, canine) and incubation temperatures (37 °C, 38 °C, 39 °C). Bars represent the proportion (%) of strains within each group. The number of strains (n) and their percentage are indicated within the bars. (**B**) Biofilm formation quantified by absorbance at 570 nm after 24 h of incubation at 37 °C, 38 °C, and 39 °C. Statistical significance was determined by two-way ANOVA followed by Tukey’s post hoc test. Differences were considered statistically significant at *p* < 0.05 [* *p* < 0.05; ** *p* < 0.01].

Biofilm-forming capacity was assessed by measuring absorbance at 570 nm following 24 h of incubation at 37 °C, 38 °C, and 39 °C. All isolates were classified as at least weak or moderate biofilm producers. At 37 °C, 70.5% (43/61) of the strains exceeded the threshold for strong biofilm formation, while this number decreased to 50.8% (31/61) and 52.5% (32/61) at 38 °C and 39 °C, respectively. *S*. *saprophyticus* strains from healthy dogs formed strong biofilms at all three temperatures. Over 60% of the human-derived strains exhibited strong biofilm-forming capacity at 37 °C, while at 38 °Cand 39 °C, this proportion decreased to approximately 52% (Figure 3). Detailed data are provided in Table S3 in the Supplementary Materials.

To assess the influence of incubation temperature and host origin on biofilm formation, a two-way ANOVA was conducted using absorbance values at 570 nm measured for all *S*. *saprophyticus* strains (n = 61) at all three temperatures. The analysis revealed a statistically significant effect of incubation temperature on biofilm formation (F(2.174) = 8.72, *p* = 0.0002), indicating that temperature has a substantial impact on the extent of biofilm production. In contrast, no statistically significant effect of host origin was observed (F(2.174) = 1.44, *p* = 0.2408), nor was there a significant interaction between temperature and host origin (F(4.174) = 0.37, *p* = 0.8327). Tukey’s post hoc HSD test showed that the strains incubated at 37 °C exhibited significantly higher biofilm production compared to those at 38 °C (*p* = 0.0002) and 39 °C (*p* = 0.0186), while no significant difference was observed between 38 °C and 39 °C (*p* = 0.3000). These findings indicate that 37 °C is the optimal temperature for strong biofilm formation by *S*. *saprophyticus* strains, regardless of their host origin.

### 2.5. Concordance Between Phenotypic and Genotypic Resistance

To evaluate the agreement between phenotypic antimicrobial susceptibility and the presence of corresponding resistance genes, statistical analyses were performed using McNemar’s test and Cohen’s kappa coefficient. Overall, the concordance between genotypes was variable and, in many cases, low. McNemar’s test revealed statistically significant discordance (*p* < 0.05) for several gene–antibiotic combinations, including *blaZ*/ampicillin; *blaZ*/penicillin; *blaZ*/amoxicillin–clavulanic acid; *mecA*/oxacillin; *ermB*/erythromycin; *tetM*/tetracycline; *vanB*/teicoplanin; and *ac*(*6*′)*-Ie-aph*(*2*″)*-Ia*/gentamicin. The corresponding Cohen’s kappa coefficients for these pairs mostly ranged from 0.00 to 0.20, indicating no or very slight agreement. In some instances, negative kappa values were obtained, suggesting an agreement worse than expected by chance. For several combinations, kappa could not be calculated due to a lack of variability in the dataset (marked as “n/a”). These findings highlight that the mere presence of a resistance gene does not always translate into phenotypic resistance detectable by microbiological methods. Such discrepancies may result from gene expression variability, regulatory mechanism, or other non-genetic resistance factors. A summary of the most relevant results is presented in the Table 3 below. Complete phenotypic and genotypic resistance profiles are provided in the Supplementary Materials in Tables S1, S2 and S4. A full statistical comparison is provided in Table S5.

### 2.6. Analysis of Pathogenicity Tests on Galleria mellonella Larvae Model

**Figure 4:**
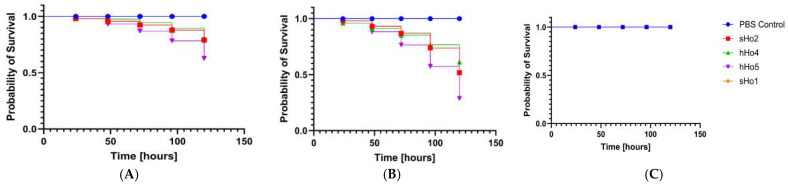
Survival curves of larvae (n = 4) following injection with *S*. *saprophyticus* strains. Larval survival was monitored for 120 h. The negative control group of larvae were injected with sterile phosphate-buffered saline (PBS). (**A**) Inoculum density OD_600_ = 0.5, (**B**) inoculum density OD_600_ = 0.1, (**C**) inoculum density OD_600_ = 0.01. Each curve represents survival of larvae (*G*. *mellonella*) infected with human-derived *S*. *saprophyticus* strains.

**Figure 5:**
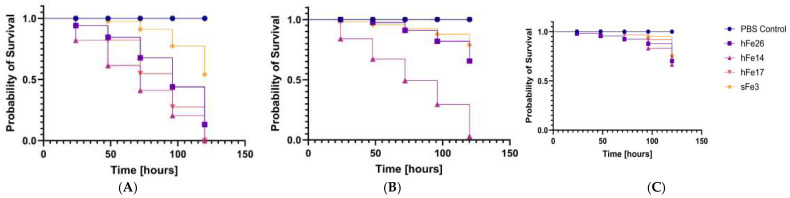
Survival curves of larvae (n = 4) following injection with *S*. *saprophyticus* strains. Larval survival was monitored for 120 h. The negative control group of larvae were injected with sterile phosphate-buffered saline (PBS). (**A**) Inoculum density OD_600_ = 0.5, (**B**) inoculum density OD_600_ = 0.1, (**C**) inoculum density OD_600_ = 0.01. Each curve represents survival of larvae (*G*. *mellonella*) infected with feline-derived *S*. *saprophyticus* strains.

**Figure 6:**
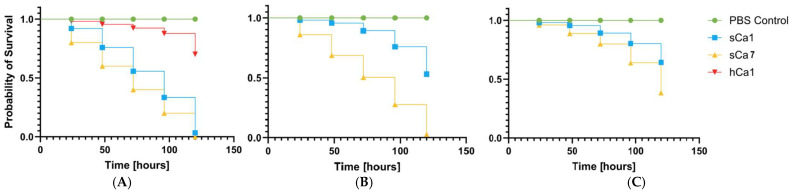
Survival curves of larvae (n = 3) following injection with *S*. *saprophyticus* strains. Larval survival was monitored for 120 h. The negative control group of larvae were injected with sterile phosphate-buffered saline (PBS). (**A**) Inoculum density OD_600_ = 0.5, (**B**) inoculum density OD_600_ = 0.1, (**C**) inoculum density OD_600_ = 0.01. Each curve represents survival of larvae (*G*. *mellonella*) infected with canine-derived *S*. *saprophyticus* strains.

All tested *S*. *saprophyticus* strains exhibited significant pathogenicity in the *Galleria mellonella* (*G*. *mellonella*) infection model. Virulence was strongly dependent on the bacterial inoculum dose. The highest larval mortality was observed at higher inoculum densities (OD_600_ = 0.5 and 0.1), while the lowest concentration (OD_600_ = 0.01) generally did not result in significant reductions in survival. Notably, feline isolate hFe17 and canine isolate sCa7 exhibited particularly high virulence, causing substantial larval mortality at all tested concentrations, indicating their potentially heightened clinical relevance. Human-derived strains were also pathogenic but displayed a clearly dependent virulence pattern. The variation in pathogenic potential among strains of different host origin suggests the presence of host-specific adaptations. These findings are presented in Figure 4A–C, Figure 5A–C and Figure 6A–C. The following strains were used in the *G*. *mellonella* infection model: sHo2, hHo4, sHo5, sHo1, hFe26, hFe14, hFe17, sFe3, sCa1, sCa7, and hCa1. These strains are marked in green in the Supplementary Materials in Tables S1 and S2 (phenotypic resistance profiles) and Table S4 (genotypic resistance profiles).

## 3. Discussion

*Staphylococcus saprophyticus* is uncommonly found in companion animals but is a significant cause of urinary tract infections, particularly cystitis, in humans [6]. Although direct evidence of transmission between humans and companion animals has not yet been clearly demonstrated, the possibility of such transmission exists and should be considered in epidemiological analyses [42]. Many studies highlight that, despite the increasing clinical importance of CoNS as causative agents of infections, their identification in routine clinical diagnostics is most often limited to the species level. This is primarily due to the fact that more detailed differentiation methods, such as advanced biochemical tests or molecular analyses, are often too time-consuming, expensive, or insufficiently precise for widespread implementation [1,43,44,45].

In recent years the use of MALDI-TOF MS has been described as a rapid and effective method for bacterial identification, which was confirmed in epidemiological studies conducted by Mlaga et al. during an outbreak of urinary tract infections caused by *S*. *saprophyticus* [46]. Our findings also support the high reliability of this method—it proved to be fast, sensitive and highly reproducible, with spectral profiles fully consistent with the results of PCR targeting the species-specific gene *hrcA* [36]. Moreover, cluster analysis based on MALDI-TOF MS spectra revealed considerable intraspecies diversity among *S*. *saprophyticus* isolates, consistent with previous reports on the high genomic plasticity of the species [46,47,48]. The highest degree of spectral homogeneity was observed among isolates obtained from healthy cats; however, even within this group, the strains were dispersed across multiple clusters. This may indicate the existence of subpopulations adapted to specific hosts and suggest the potential for dynamic population shifts as part of the adaptive strategy of the species for colonizing different hosts [49,50,51].

Phenotypic antimicrobial susceptibility testing using the disk diffusion method revealed high levels of resistance, particularly to erythromycin (47.54%, 29/61). Among the isolates obtained from dogs, an exceptionally high level of resistance to erythromycin (100.0%, 8/8) and clindamycin (50.5%, 4/8) was observed. This may reflect a species-specific selective pressure associated with routine veterinary therapeutic practices, but the limited sample size of the canine group may partially account for the high observed resistance rate. Nevertheless, these findings underscore the need for continued and systematic monitoring of resistance to this antibiotic in strains obtained from dogs. Similar patterns of high resistance to erythromycin and clindamycin in *S*. *saprophyticus* have been reported previously, including findings by Rafie et al. who observed erythromycin resistance in 51.4% of isolates [52]. Khan et al. reported that 91% of *S*. *saprophyticus* strains exhibited resistance to penicillin, a markedly higher rate compared to our findings [53]. In our study, penicillin resistance was observed primarily among human-derived strains, particularly those obtained from healthy individuals (35.71%, 5/14). Among strains derived from animals, no resistance was detected in canine strains, while in the feline population, it was observed only in healthy cats (24.14%, 7/29). In contrast, Marepalli et al. documented ampicillin resistance in approximately 50.0% of isolates, which closely mirrors our results [54]. In our data, ampicillin resistance was highest among human-derived strains, with similar rates found in strains from healthy individuals (50.0%, 7/14) and those with clinical symptoms (42.86%, 3/7). Oxacillin resistance in our study reached 42.62% (26/61), with comparable rates between human- and animal-derived strains. Reports in the literature also describe a wide range of resistance levels to this antibiotic, ranging from 76.2% [1] to as high as 98.2%, based on zone-of-inhibition analysis [55]. These findings confirm that the resistance of *S*. *saprophyticus* to many antibiotics (erythromycin, clindamycin, oxacillin, penicillin) is not a local phenomenon but rather a widespread and clinically relevant issue.

Additionally, the use of MIC determination for the selected antibiotics allowed for a more precise assessment of actual resistance levels. In the case of oxacillin and tetracycline, there was high concordance between the MIC and disk diffusion results (difference ≤ 3%). However, for erythromycin and clindamycin, significant discrepancies were noted—the disk diffusion method resistance rates, by 45.9% (28/61) and 14.75% (9/61), were lower than those obtained in the broth microdilution method, at 78.69% (48/61) and 44.26% (27/61), respectively. This may be attributed to the classification strategy applied: isolates were considered susceptible if bacterial growth did not clearly extend beyond the edge of the inhibition zone, even when growth reached its boundary. While this approach aimed to avoid overinterpretation and the risk of falsely classifying strains as resistant, it led to an underestimation of the number of truly resistant strains compared to MIC references values. It is worth noting that similar interpretive challenges, particularly for erythromycin and oxacillin in staphylococci, have been previously reported [56]. In the subsequent part of the study the MBC/MIC ratio was assessed for the selected antibiotics to identify potential mechanism of antibiotic tolerance. The highest frequency of elevated MBC/MIC ratios (log_2_[MBC/MIC] ≥ 4) was observed for tetracycline, affecting 49.2% (30/61). A high MBC/MIC ratio may indicate the presence of tolerance mechanisms, such as biofilm formation, active drug efflux systems, or decreased cell membrane permeability. Similar findings have been documented in earlier research [57,58]. The smallest differences between MBC/MIC values were recorded for vancomycin and teicoplanin, both of which demonstrated complete efficacy against all tested strains in MIC-based assessments. These findings are consistent with earlier studies reporting that *S. saprophyticus* remains susceptible to glycopeptides, particularly vancomycin, although some strains may exhibit intermediate susceptibility to teicoplanin (MIC range 2–8 µg/mL) [59]. Our results reaffirm the therapeutic utility of glycopeptides against this species, especially in the context of increasing resistance to first-line antibiotics such as macrolides, tetracyclines, and β-lactams. Importantly, strains obtained from healthy animals exhibited lower and more stable log_2_(MBC/MIC) ratios, which may reflect selective pressure in non-clinical environments and a potentially limited expression of tolerance mechanisms in these strains.

All analyzed *S*. *saprophyticus* strains carried the *blaZ* and *mecA* genes, which are associated with widespread resistance to β-lactam antibiotics. The simultaneous presence of both determinants indicates the coexistence of enzymatic inactivation mechanisms (β-lactamase production) and altered penicillin-binding proteins (PBP2a) characteristic of methicillin-resistant coagulase-negative staphylococci (MRCoNS). These results are consistent with previous studies reporting the detection of *blaZ* and *mecA* in *S*. *saprophyticus* strains isolated from both humans and animals [60]. Genes associated with MLSb resistance, *ermA* and *ermB*, were particularly prevalent among the human-derived isolates, which may reflect selective pressure resulting from the frequent use of macrolides in the treatment of urinary tract infections. The presence of these genes in *S*. *saprophyticus* has also been documented in the context of urological infections [61]. Determinants of tetracycline resistance *tetM* and *tetK* were dominant among the strains from infected humans and cats, possibly indicating convergent selection pressures or similar levels of exposure to tetracyclines in these populations. Their presence has previously been documented in CoNS strains isolated from bovine mastitis cases, confirming the widespread distribution of these genes across different environments [62,63]. Particular attention should be given to the *fusB* and *mupA* genes, which are responsible for resistance to fusidic acid and mupirocin. The *fusB* gene was detected in 13.79% (4/29) and *mupA* in 6.9% (2/29) of strains, both exclusively among the strains derived from cats. In all cases, phenotypic resistance confirmed by the disk diffusion method corresponded with the presence of the respective gene, suggesting active expression. Notably, the highest levels of phenotypic resistance to fusidic acid were observed in isolates obtained from diseased humans despite the absence of resistance genes related to this antibiotic. This may indicate the involvement of alternative, as-yet-unidentified resistance mechanisms. Considering the frequent use of fusidic acid in human dermatology, particularly for the topical treatment of skin infections, this observation may have important clinical relevance [64]. Although the total number of *mupA*-positive cases was low (2/61), the presence of these genes in commensal strains isolated from companion animals may indicate a reservoir of resistance determinants that are rarely found in the human population. The detection of such genes, even in individual cases, highlights the importance of continued surveillance of this bacterial group, especially in the context of close human–pet interactions [65,66]. These findings point to a significant dissemination of resistance genes within the *S*. *saprophyticus* species, regardless of the isolation source, and highlight the need for continued monitoring of this organism as a potential reservoir of clinically and epidemiologically relevant resistance determinants. However, this study focused solely on the presence of major resistance genes and did not investigate allelic variants or emerging mechanisms such as efflux pumps or chromosomal mutations (e.g., in 23S rRNA or *rpoB*). Future studies incorporating whole-genome sequencing or transcriptomic approaches are warranted to better elucidate the full spectrum of resistance determinants in *S*. *saprophyticus*. Importantly, genotypic resistance did not always correlate with phenotypic resistance, indicating notable discrepancies between genotypic and phenotypic profiles across various antibiotics.

MDR, defined as resistance to three or more classes of antibiotics [41], was observed in 38.1% of the human-derived strains (8/21) and 32.5% of the animal-derived strains (13/40). Although the proportion was slightly higher among the human isolates, the difference was not statistically significant, suggesting that MDR *S*. *saprophyticus* strains occur in both human and animal populations. This finding may indicate the widespread distribution of resistance mechanisms across environments and highlights the potential role of animal-derived strains in resistance gene dissemination. The presence of MDR strains among healthy companion animals further emphasizes the importance of including these populations in antimicrobial resistance surveillance programs, particularly given their close contact with humans. The phenotypic resistance profile was further supported by genotypic analysis. All examined *S*. *saprophyticus* strains were susceptible to glycopeptides (vancomycin and teicoplanin), in agreement with MIC data and consistent with previous reports describing CoNS susceptibility to this antibiotic group [1,67].

In the analysis of genotype/phenotype (gene/antibiotic) resistance concordance using McNemar’s test and Cohen’s kappa, discrepancies between the presence of resistance genes and phenotypic resistance may arise from several factors. Among the most important are a lack of gene expression (e.g., due to regulatory mutations), the presence of pseudogenes, alternative resistance mechanisms, as well as limitations in the sensitivity of phenotypic tests. Moreover, interpretation of the results may be hindered in cases of a low number of isolates or their uneven distribution across the studied groups. Such discrepancies are well known and have also been reported in other studies on resistance in bacteria of the genera *Staphylococcus*, *Enterococcus*, and *Enterobacterales*.

High resistance rates of *S*. *saprophyticus* isolates to erythromycin, clindamycin, and oxacillin significantly limit the options for empirical treatment of urinary tract and skin infections. The absence of resistance to vancomycin, teicoplanin, and linezolid suggests that these antibiotics may remain effective in more cases, although their use should be reserved for targeted therapy [68,69]. In the empirical treatment of uncomplicated urinary tract infections in humans, the most recommended antibiotics are nitrofurantoin, fosfomycin, and trimethoprim with sulfamethoxazole [68]. In companion animals such as dogs and cats, fluoroquinolones, amoxicillin–clavulanic acid, and trimethoprim with sulfamethoxazole are frequently used [70]. For skin infections in humans, clindamycin, doxycycline, and cephalexin are commonly prescribed [69], while in animals, cephalexin, clindamycin, and fluoroquinolones are preferred [71]. Our findings partially align with these recommendations—high resistance to erythromycin and clindamycin may reflect their frequent use, suggesting the influence of selective pressure. The presence of resistant strains in both humans and animals, including healthy individuals, indicates a potential risk of interspecies transmission. In the context of rising resistance levels and the occurrence of multidrug-resistant strains, it is essential to promote rational antibiotic use in both groups of patients and systematically monitor local resistance profiles.

Both the biofilm formation assays and the growth curve analysis consistently identified 37 °C as the optimal temperature for *S*. *saprophyticus*, highlighting the strong physiological adaptation of the species to host-like conditions. At this temperature, the highest proportion of strong biofilm producers was recorded, and the strains exhibited the fastest growth rates and highest OD_600_ plateau values. In contrast, increasing the incubation temperature to 38 °C and 39 °C led to a general decline in both biofilm-forming capacity and growth efficiency, suggesting a degree of temperature sensitivity in the regulatory mechanisms governing these processes. This trend was particularly evident among animal-derived strains, which showed reduced performance in both traits at elevated temperatures. The convergence of these findings underscore the potential role of temperature as a key environmental signal influencing not only bacterial metabolism but also the expression of virulence-associated phenotypes such as biofilm formation. Thus, 37 °C appears to represent not only the physiological but also the functional optimum for *S*. *saprophyticus* survival and persistence within the host [31,72]. Physiological body temperatures in cats (approximately 38–39.2 °C) and dogs (around 37.5–39 °C) are consistently higher than the typical human core temperature of 37 °C. This thermal difference could represent a significant physiological barrier, potentially explaining the relatively lower rates of *S*. *saprophyticus* isolation from companion animals compared to humans.

This study represents the first attempt to assess the virulence of *S*. *saprophyticus* using the *G*. *mellonella* larvae infection model [73]. All tested strains demonstrated pathogenic potential in this model, with the highest larval mortality observed for isolates hFe14 and sCa7. The fastest growing strain, hFe26, also exhibited high virulence; however, no clear correlation was found between growth rate and pathogenicity. These results suggest that additional virulence factors, such as biofilm formation or toxin production, may also play a significant role [1,74]. The *G*. *mellonella* model has been widely used as a cost-effective, ethically advantageous alternative to mammalian infection models. Several studies have shown that results obtained in *G*. *mellonella* correlate well with those from murine models, including infections caused by coagulase-negative staphylococci [73]. In the case of *Staphylococcus aureus* and *Staphylococcus epidermidis*, this model has provided comparable insight into virulence mechanism and host–pathogen interactions [75,76]. Additionally, a murine model of urinary tract infection (UTI) caused by *S*. *saprophyticus* has been previously described. In this model, colonization of the kidneys and subsequent infection of the bladder were observed, with the surface proteins Ssp and Sdrl shown to play significant roles in virulence [18]. While the present study did not include an assessment of toxin production or virulence gene expression, these aspects will be the subject of future investigation. Interestingly, the most virulent strains, such as hFe14 and sCa7, displayed multidrug resistance phenotypes and carried several resistance genes (e.g., *blaZ*, *mecA*, *ermB*, *tetM*), which may suggest a potential link between antibiotic resistance and virulence in *S*. *saprophyticus*. These findings reinforce the notion that coagulase-negative staphylococci, including *S*. *saprophyticus*, possess significant pathogenic potential and the ability to develop multidrug resistance.

Interestingly, the most virulent strains, such as hFe14 and sCa7, exhibited multidrug resistance phenotypes and harbored multiple resistance genes (e.g., *blaZ*, *mecA*, *ermB*, *tetM*), suggesting a potential link between antimicrobial resistance and virulence in *S*. *saprophyticus*. The results of this study confirm that CoNS, including *S*. *saprophyticus*, possess significant pathogenic potential and the capacity to develop multidrug resistance [1]. Accurate species identification and resistance profiling remain essential for effective treatment and for reducing the risk of interspecies transmission. Given the high proportion of MDR strains (39.34%, 21/61), routine antimicrobial susceptibility testing should be a standard component of microbiological diagnostics [74].

It is worth emphasizing that *S*. *saprophyticus* and other CoNS were for many years routinely regarded as contaminants in clinical or environmental samples. However, numerous studies have demonstrated that such isolates should not be automatically classified as contaminants, particularly considering their documented ability to form biofilms, carry resistance genes, and participate in opportunistic infections. Importantly, the presence of strains from companion animals exhibiting concordant phenotypic and genotypic resistance highlights the need to consider this group as a potential reservoir of resistant microorganisms within antimicrobial resistance monitoring programs. However, the relatively small size of the animal subgroups in this study limits the strength of direct comparisons, and further investigations on larger cohorts are needed to better understand and monitor these trends.

## 4. Materials and Methods

### 4.1. S. saprophyticus Strains Collection

The strain collection for this study consisted of 61 *S*. *saprophyticus* deposited in the years 2013–2022 during research carried out in the Department of Epizootiology and the Clinic of Birds and Exotic Animals of the Faculty of Veterinary Medicine, University of Environmental and Life Sciences in Wroclaw. Sampling and the necessary approvals of ethics committees were described earlier [25,77]. The strains were isolated from swabs taken from two groups of patients: healthy dogs, cats, and humans without any symptoms of the disease; and sick dogs, cats, and humans with symptoms of disease (coughing, discharge from conjunctival sac, or skin lesions). In the current study, 21 samples from humans (n = 14 healthy, n = 7 sick) and 40 samples from dogs and cats (n = 30 healthy, n = 10 sick) were investigated. All bacterial strains were stored at −80 °C in Brain Heart Infusion (BHI) broth (OXOID Ltd., Basingstone, UK) containing 40% (*v*/*v*) glycerol (Thermo Fischer Scientific, Kandel, Germany). Detailed information on sampling sites and group sizes is presented in Table 4.

### 4.2. Identification of S. saprophyticus

All bacterial strains were cultured on Mannitol Salt Agar (OXOID Ltd., Bansingston, UK) and Columbia Agar (OXOID Ltd., Bansingston, UK) supplemented with 5% sheep blood for 24 h at 37 °C. Primary identification of the strains as *Staphylococcus* spp. was conducted using colony morphology and tube test coagulase (Immunolab, Gdańsk, Poland). Then, bacterial species were identified using a Bruker ultrafle Xtreme MALDI-TOF mass spectrometer (Bruker Daltonics, Bremen, Germany). To ensure reliability, each strain underwent duplication with two technical replicates prepared per extract. Sample preparation involved ethanol/formic acid extraction: single colonies were resuspended in water, treated with ethanol (Sigma Aldrich, Saint Louis, MO, USA), and centrifuged. The resulting pellet was then processed with 70% formic acid (Sigma Aldrich, Saint Louis, MO, USA) and acetonitrile (Honeywell, Charlotte, NC, USA) followed by a second centrifugation. A total of 1 µL of the supernatant was spotted onto a MALDI target plate, dried, and overlaid with 1 µL of a 10 mg/mL HCCA (alpha-cyano-4-hydroxycinnamic acid) matrix solution (acetonitrile–water–TFA, 50:47.5:3.5) (Honeywell, Charlotte, NC, USA). To simplify the collected data, the dendrogram and principal component analysis (PCA) were performed based on the MALDI-TOF spectral peak profiles [78].

### 4.3. Detection of hrcA and Resistance Genes Using PCR-Based Genotypic Analysis

Additional confirmation of species affiliation and identification of the presence of genes that encode resistance to key antibiotic classes were performed using conventional PCR. Genomic DNA from all strains was extracted using a Genomic Mini AX Staphylococcus Spin kit (A&A Biotechnology, Gdansk, Poland). The analysis was focused on the following genes: *hrcA* encoding a heat shock repressor for species identification; *blaZ* encoding a β-lactamase responsible for penicillin resistance; *mecA* and *mecC*, which encode penicillin-binding proteins (PBP2a/PBP2c) associated with methicillin resistance; *ermA*, *ermB*, and *ermC*, which encode methyltransferases involved in macrolide–lincosamide–streptogramin B resistance; and the tetracycline resistance genes *tetK* and *tetL* (efflux pump genes), as well as *tetM* and *tetO*, which encode ribosomal protection proteins. Furthermore, glycopeptide resistance genes *vanA* and *vanB* were included, along with *mupA*, *aac*(*6*′)*-Ie-aph*(*2*″)*-Ia* (encoding aminoglycoside acetyltransferase), and *fusB* (associated with fusidic acid resistance) [36,79,80,81,82,83,84]. Each PCR amplification was performed in a 25 µL total reaction volume, consisting of 1 µL of DNA template, gene-specific primer pair at a 0.2 µM final concentration (Eurofins Genomics Germany GmbH, Ebersberg bei München, Germany), 0.2 mM deoxyribonucleotide triphosphate mix (dNTPs) (Thermo Fisher Scientific, Waltham, MA, USA), 2.5 µL of 10× DreamTaq Green Buffer, and 1 U of DreamTaq Green Polymerase (Thermo Fisher Scientific, Waltham, MA, USA). The thermal cycling parameters included an initial denaturation step at 94 °C for 3 min, followed by 35 cycles of denaturation at 94 °C for 30 s, annealing at temperatures specific for each primer pair 52–60 °C for 30 s, and extension at 72 °C for 1 min, with a final extension step at 72 °C for 5 min. The PCR products were separated by electrophoresis at 100 V on a 2% agarose gel stained with Midori Green DNA Stain (Nippon Genetics Europe GmbH, Dueren, Germany).

### 4.4. Antimicrobial Phenotypic Testing

The disk diffusion method was used to test phenotypic antibiotic resistance for all isolates. Antimicrobials disks used in the study were (μg/disk) as follows: amoxicillin with clavulanic acid (30), ampicillin (10), ciprofloxacin (5), chloramphenicol (30), clindamycin (2), erythromycin (15), fusidic acid (10), gentamicin (10), linezolid (30), oxacillin (5), mupirocin (200), penicillin G (10), rifampicin (5), tetracycline (30), tigecycline (15), and trimethoprim/sulfamethoxazole (1.25/23.75) (Liofilchem, Abruzzi, Italy) and marbofloxacin (5) (MASTDISCS^®^ AST (Mast Group Ltd., Liverpool, UK)). Positive control for the disk diffusion method was performed using the reference strain *Staphylococcus aureus* ATCC 25923, as recommended by the CLSI/EUCAST guidelines.

The interpretation of the antimicrobial susceptibility results was performed according to the Clinical and Laboratory Standards Institute guidelines and European Committee on Antimicrobial Susceptibility Testing (EUCAST). For human isolates, breakpoints defined in CLSI M100, 34th Edition (2024), were used. For isolates obtained from dogs and cats, veterinary-specific breakpoints provided in CLSI VET01S, 7th Edition (2024), were applied [38,39,40]. For antibiotics lacking official interpretive criteria (breakpoints) for *S*. *saprophyticus* according to the CLSI, VET CLSI, and EUCAST guidelines, breakpoints established for other staphylococcal species, primarily *S*. *aureus* were applied. For ampicillin, interpretive criteria from the pre-2015 CLSI guidelines were used, when specific breakpoints were still available. In the case of marbofloxacin, interpretation was based on data from a scientific publication [85], which provided zone diameter values derived from disk diffusion testing. In the case of mupirocin and fusidic acid, official breakpoints for *S*. *saprophyticus* are not provided by either CLSI M100 or EUCAST. EUCAST recommends the use of these agents mainly for *S*. *aureus* in the context of decolonization, and CLSI does not include these agents in its interpretive tables for *S*. *saprophyticus*. Therefore, breakpoints established for other staphylococcal species were applied. A similar approach was adopted for enrofloxacin and ciprofloxacin, for which interpretation was based on guidelines for other *Staphylococcus* spp. due to absence of specific breakpoints for *S*. *saprophyticus*. This approach enabled the interpretation of results for all 17 tested antimicrobial agents, using a combination of official sources (CLSI M100, VET01S, EUCAST) and adapted interpretive strategies when spices-specific criteria were unavailable.

MIC values were determined using the broth microdilution method in 96-well microtiter plates. The bacterial isolates were inoculated into Mueller–Hinton broth (Oxoid, Basingstoke, UK) containing serial dilutions of antibiotics prepared in sterile distilled water. Final concentrations of the tested antibiotics were as follows: for oxacillin, clindamycin, and tetracycline, 16; 8; 4; 2; 1; 0.5; 0.25; 0.125; 0.0625; and 0.03125 µg/mL; for teicoplanin and vancomycin, 128; 64; 32; 16; 8; 4; 2; 1; and 0.5 µg/mL; and for erythromycin, 32; 16; 8; 4; 2; 1; 0.5; 0.25; 0.125; and 0.0625 µg/mL (TOKU-E, Gent, Belgium) [38,39,40]. Each row of the plate corresponded to a single isolate, and tests were performed separately for each antibiotic. Each plate included a growth control (K+) containing broth and bacterial inoculum (without antibiotic) and a sterility control (K-) containing broth and antibiotic but no bacteria. *S*. *aureus* ATCC 29213 was used as the reference control strain. If the highest tested concentration of an antibiotic was insufficient to inhibit bacterial growth evidenced by turbidity in all wells, the assay was repeated using an extended dilution series, shifted one level higher. This adjustment was applied to selected isolates, except in the case of vancomycin and teicoplanin, for which the initial concentration range proved sufficient. The inoculum with an optical density of 0.5 McFarland was prepared from a fresh 24 h culture on blood agar. Plates were incubated aerobically at 37 °C for 24 h. The MIC was defined as the lowest antibiotic concentration at which no visible bacterial growth (no turbidity) was observed. The minimum bactericidal concentration (MBC) was determined by subculturing 10 µL from each well without visible growth on Brain Heart Infusion Agar (BHA, Oxoid, Basingstoke, UK). Plates were incubated at 37 °C for 24 h. The MBC was defined as the lowest concentration of the antibiotic that resulted in no colony growth, indicating ≥99.9% bacterial killing relative to the original inoculum.

To compare the minimum inhibitory concentration (MIC) and the minimum bactericidal concentration (MBC) for each bacterial isolate and antibiotic tested, the MBC/MIC ratio was calculated. These values were subsequently transformed to a base-2 logarithmic scale using the following formula: log_2_(MBC/MIC). The use of a logarithmic transformation is justified by the exponential nature of MIC and MBC values, which commonly follow two-fold dilution series (e.g., 0.125, 0.25, 0.5, 1, 2, 4, etc.). The log_2_ scale enables a proportional and statistically comparable evaluation of the differences between MIC and MBC values. A log_2_(MBC/MIC) value of 0 indicates complete equivalence between the MIC and MBC, suggesting a bactericidal effect. A value of 1 indicates that the MBC is twice as high as the MIC, while a value of 2 corresponds to a fourfold difference, and so on. This analysis was performed for six antibiotics: vancomycin, teicoplanin, tetracycline, oxacillin, erythromycin, and clindamycin.

### 4.5. Bacterial Growth Curves and Biofilm Formation

Growth profiles of chosen *S*. *saprophyticus* strains (sCa1, sCa7, hCa1, hFe26, hFe14, hFe17, sFe3, sHo2, hHo4, hHo5, sHo1—strains were selected based on the highest number of resistance genes and the highest phenotypic antibiotic resistances in each of the possible groups tested) and reference strains (*S*. *aureus* ATCC 43300, *S*. *epidermidis* PCM 1869, *S*. *saprophyticus* PCM 2109) were assessed using Brain Heart Infusion (BHI) broth (Oxoid, Basingstoke, UK). Overnight cultures were diluted in 5 mL of BHI to an optical density (OD) of approximately 0.1 at 600 nm (Eppendorf BioPhotometer 6131, Hamburg, Germany). Subsequently, 500 µL of each diluted culture was added to triplicate wells of a 48-well plate and incubated with automated shaking at 37 °C, 38 °C, and 39 °C for 48 h. Absorbance at 600 nm was measured using an automated absorbance reader (Tecan Infinite 200 Pro M Nano, Männedorf, Switzerland). Biofilm production by *S*. *saprophyticus* strains was assessed using the microtiter plate (MTP) method with crystal violet staining [86]. The *S*. *aureus* ATCC 29213 strain was used as a positive control, while the negative control consisted of sterile Tryptone Soya Broth (TSB, Oxoid, Basingstoke, UK). Following incubation and staining, absorbance was measured at 570 nm (A570) using a microplate reader (Tecan Infinite Pro, Männedorf, Switzerland). The cut-off value (OD) for determining biofilm formation was defined as the mean absorbance of the negative control plus two standard deviations. Biofilm-forming ability was classified based on the relationship between A570 values and the OD threshold as follows: weak biofilm producers: OD < A570 ≤ 2 × OD; moderate biofilm producers: 2 × OD < A570 ≤ 4 × OD; strong biofilm producers: A570 > 4 × OD.

### 4.6. Pathogenicity Tests on Galleria mellonella Larvae Model

Overnight cultures of selected *S*. *saprophyticus* strains (n = 11; sCa1, sCa7, hCa1, hFe26, hFe14, hFe17, sFe3, sHo2, hHo4, hHo5, sHo1) were centrifuged at 3500 RPM. The resulting bacterial pellets were washed three times with sterile PBS buffer (Argenta, Poznan, Poland) and resuspended to achieve optical densities of OD_600_ = 0.5, OD_600_ = 0.1, and OD_600_ = 0.01. The larvae used in the experiment were obtained from a self-maintained culture. Larvae weighing 300 ± 30 mg were selected and placed in groups of 10 (n = 10) on sterile Petri dishes. Each larva was injected with 10 μL of bacterial suspension directly into the hemocoel using a Hamilton Bonaduz 100 μL microliter syringe (Hamilton Bonaduz AG, Bonaduz, Switzerland). For the negative control, larvae (n = 10) were injected with 10 μL of sterile PBS buffer. The experiment was repeated at least three times. Larval survival was monitored over 120 h at 37 °C post-injection, with the number of live and dead larvae recorded. Larvae were considered dead when they appeared darkened and unresponsive to touch. The data were analyzed statistically using the GraphPad Prism 10.4.1 software with the Kaplan–Meier algorithm [73]. Survival curves were generated based on the results.

### 4.7. Statistical Methods

The influence of incubation temperature and host origin on biofilm formation was analyzed using a two-way analysis of variance (ANOVA) with interaction. Temperature and host origin were included as fixed factors, and absorbance A570 nm was used as the dependent variable. Post hoc pairwise comparisons were performer using Tukey’s Honestly Significant Difference (HSD) test to identify significant differences between group means. A *p*-value < 0.05 was considered statistically significant. Statistical analysis was performer using Statistica 13 (TIBCO Software Inc., Tulsa, OK, USA). To assess the correlation between the presence of resistance genes and phenotypic antibiotic resistance, McNemar’s test and Cohen’s kappa were applied using Statistica 13 (TIBCO Software Inc., Tulsa, OK, USA).

The interactive plots included in the Supplementary Materials were generated using Python (v3.10) and Plotly (v5.x) libraries. The HTML files allow for dynamic exploration of the results.

## Figures and Tables

**Table 1:** Resistance (%) to selected antibiotics in *S*. *saprophyticus* strains from humans and animals determined by the disk diffusion method. A bar chart compares resistance to each antibiotic across the studied groups.

Antimicrobial Agents Evaluated																	
Group	AMP	AMC	MUP	P	FD	MAR	CIP	OX	LZD	DA	E	C	RD	CN	SXT	TGC	TET
Healthy humans (n = 14)	7 (50.0%)	2 (14.29%)	0 (0.0%)	5 (35.71%)	4 (28.57%)	1 (7.14%)	0 (0.0%)	6 (42.86%)	0 (0.0%)	1 (7.14%)	7 (50.0%)	2 (14.29%)	0 (0.0%)	1 (7.14%)	0 (0.0%)	1 (7.14%)	0 (0.0%)
Sick humans (n = 7)	3 (42.86%)	0 (0.0%)	0 (0.0%)	1 (14.29%)	3 (42.86%)	0 (0.0%)	1 (14.29%)	3 (42.86%)	0 (0.0%)	0 (0.0%)	4 (57.14%)	0 (0.0%)	0 (0.0%)	0 (0.0%)	0 (0.0%)	0 (0.0%)	1 (14.29%)
Healthy cats (n = 29)	6 (20.69%)	2 (6.9%)	2 (6.9%)	7 (24.14%)	5 (17.24%)	2 (6.9%)	4 (13.79%)	9 (31.03%)	1 (3.45%)	3 (10.34%)	9 (31.03%)	2 (6.9%)	1 (3.45%)	2 (6.9%)	0 (0.0%)	0 (0.0%)	5 (17.24%)
Sick cats (n = 3)	0 (0.0%)	0 (0.0%)	0 (0.0%)	0 (0.0%)	1 (33.33%)	0 (0.0%)	0 (0.0%)	2 (66.66%)	0 (0.0%)	1 (33.33%)	1 (33.33%)	0 (0.0%)	0 (0.0%)	0 (0.0%)	0 (0.0%)	0 (0.0%)	1 (33.3%)
Healthy dogs (n = 1)	0 (0.0%)	0 (0.0%)	0 (0.0%)	0 (0.0%)	0 (0.0%)	0 (0.0%)	0 (0.0%)	0 (0.0%)	0 (0.0%)	1 (100.0%)	1 (100.0%)	0 (0.0%)	0 (0.0%)	0 (0.0%)	0 (0.0%)	0 (0.0%)	0 (0.0%)
Sick dogs (n = 7)	1 (14.29%)	0 (0.0%)	0 (0.0%)	0 (0.0%)	2 (28.57%)	0 (0.0%)	0 (0.0%)	6 (85.71%)	0 (0.0%)	3 (42.86%)	7 (100.0%)	1 (14.29%)	0 (0.0%)	0 (0.0%)	0 (0.0%)	0 (0.0%)	1 (14.29%)
All (n = 61)	17 (27.87%)	4 (6.56%)	2 (3.28%)	13 (21.31%)	15 (24.59%)	3 (4.92%)	5 (8.20%)	26 (42.62%)	1 (1.64%)	9 (14.75%)	29 (47.54%)	5 (8.20%)	1 (1.64%)	3 (4.92%)	0 (0.0%)	1 (1.64%)	8 (13.11%)

AMP = ampicillin, AMC = amoxicillin/clavulanic acid, MUP = mupirocin, P = penicillin, FD = fusidic acid, MAR = marbofloxacin, CIP = ciprofloxacin, OX = oxacillin, LZD = linezolid, DA = clindamycin, E = erythromycin, C = chloramphenicol, RD = rifampicin, CN = gentamicin, SXT = trimethoprim/sulfamethoxazole, TGC = tigecycline, TET = tetracycline.

**Table 2:** Occurrence of selected antibiotic resistance genes among *S*. *saprophyticus* strains from humans and animals (%).

Antimicrobial Resistance Genes Tested															
Group	*blaZ*	*mecA*	*mecC*	Aac *	*ermA*	*ermB*	*ermC*	*tetK*	*tetL*	*tetM*	*tetO*	*fusB*	*vanA*	*vanB*	*mupA*
Healthy humans (n = 14)	14 (100%)	14 (100%)	0 (0%)	0 (0%)	13 (92.86%)	13 (92.86%)	0 (0%)	0 (0.0%)	10 (71.43%)	14 (100%)	0 (0%)	0 (0%)	0 (0%)	1 (7.14%)	0 (0%)
Sick humans (n = 7)	7 (100%)	7 (100%)	0 (0%)	0 (0%)	6 (85.71%)	4 (57.14%)	0 (0%)	0 (0%)	7 (100%)	7 (100%)	2 (28.57%)	0 (0%)	0 (0%)	0 (0%)	0 (0%)
Healthy cats (n = 29)	29 (100%)	29 (100%)	0 (0%)	4 (13.79%)	25 (86.21%)	20 (68.97%)	4 (13.79%)	0 (0%)	8 (27.59%)	28 (96.55%)	5 (17.24%)	4 (13.79%)	0 (0%)	0 (0%)	2 (6.9%)
Sick cats (n = 3)	3 (100%)	3 (100%)	0 (0%)	0 (0%)	3 (100%)	1 (33.3%)	0 (0%)	0 (0%)	2 (66.67%)	3 (100%)	1 (33.33%)	0 (0%)	0 (0%)	0 (0%)	0 (0%)
Healthy dogs (n = 1)	1 (100%)	1 (100%)	0 (0%)	0 (0%)	1 (100%)	0 (0%)	0 (0%)	0 (0%)	0 (0%)	1 (100%)	0 (0%)	0 (0%)	0 (0%)	0 (0%)	0 (0%)
Sick dogs (n = 7)	7 (100%)	7 (100%)	0 (0%)	0 (0%)	6 (85.71%)	3 (42.86%)	0 (0%)	0 (0%)	5 (71.43%)	7 (100%)	1 (14.29%)	0 (0%)	0 (0%)	1 (14.29%)	0 (0%)
All (n = 61)	61 (100.0%)	61 (100%)	0 (0.0%)	4 (6.56%)	54 (88.52%)	41 (67.21%)	4 (6.56%)	0 (0%)	32 (52.46%)	60 (98.36%)	11 (18.03%)	4 (6.6%)	0 (0.0%)	2 (3.28%)	2 (3.28%)

* *aac(6′)-Ie-aph(2″)-Ia*.

**Table 3:** Summary of concordance between the presence of resistance genes and phenotypic antibiotic resistance based on Mcnemar’s test and Cohen’s kappa coefficient. DD—disk diffusion; MIC—minimum inhibitory concentration; κ—Cohen’s kappa coefficient. “+” in the McNemar’s column indicates a statistically significant discordance (*p* < 0.05).

No.	Gene/Antibiotic	Agreement Type	McNemar’s *p* < 0.05	Cohen’s κ
1	*blaZ*/ampicillin	No agreement	+	0.00
2	*blaZ*/penicillin	No agreement	+	0.00
3	*blaZ*/amoxicillin–clavulanic acid	No agreement	+	0.00
4	*mecA*/oxacillin (DD)	No agreement	+	0.00
5	*ermB*/erythromycin (MIC)	Slight agreement	-	0.06
6	*tetM*/tetracycline (MIC)	Worse than chance agreement	+	−0.03
7	*vanB*/teicoplanin	Perfect agreement	-	1.00
8	*fusB*/fusidic acid	Slight agreement	+	0.12
9	*mupA*/mupirocin	Perfect agreement	-	1.00
10	*aac*(*6*′)*-Ie-aph*(*2*″)*-Ia*/gentamicin	Slight agreement	-	0.16

“+”—statistically significant difference (McNemar’s *p* < 0.05); “-”—no significant difference. Cohen’s κ: 1.00—perfect agreement, 0.00—no agreement, <0—worse than chance.

**Table 4:** The table shows a detailed breakdown of the data relating to the number of samples taken from a given location for each group of patients and volunteers.

Groups	No Data	Oral Cavity	Nostrils	Anus	Wound	Skin	Ear Canal	Conjunctival Sac	Total
Healthy humans	0	2	4	0	0	4	4	0	14
Sick humans	5	0	1	0	0	1	0	0	7
Healthy cats	0	2	6	2	0	6	5	8	29
Sick cats	0	0	3	0	0	0	0	0	3
Healthy dogs	0	0	0	0	0	0	0	1	1
Sick dogs	0	0	1	0	1	0	2	3	7
All	5	4	15	2	1	11	11	12	61

## Data Availability

Data is contained within the article and Supplementary Materials.

## Supplementary Materials

The following supporting information can be downloaded at: https://www.mdpi.com/article/10.3390/ijms26146885/s1.

## References

[1] Becker K., Heilmann C., Peters G. (2014). Coagulase-negative staphylococci. Clin. Microbiol. Rev..

[2] Podkowik M., Bania J., Schubert J., Bystroń J. (2014). Gronkowce koagulazo-ujemne: Nowe zagrożenie dla zdrowia publicznego?. Życie Weter..

[3] Huebner J., Goldmann D.A. (1999). Coagulase-negative staphylococci: Role as pathogens. Annu. Rev. Med..

[4] Heilmann C., Ziebuhr W., Becker K. (2019). Are coagulase-negative staphylococci virulent?. Clin. Microbiol. Infect..

[5] Otto M. (2004). Virulence factors of coagulase-negative staphylococci. Front. Biosci..

[6] Raz R., Colodner R., Kunin C.M. (2005). Who are you—*Staphylococcus saprophyticus*?. Clin. Infect. Dis..

[7] Rupp M.E., Soper D.E., Archer G.L. (1992). Colonization of the female genital tract with *Staphylococcus saprophyticus*. J. Clin. Microbiol..

[8] Białek B., Tyski S., Hryniewicz W., Kasprowicz A., Heczko P.B. (1990). Role of *Staphylococcus saprophyticus* in human infection. Acta Microbiol. Pol..

[9] Latham R.H., Running K., Stamm W.E. (1983). Urinary tract infections in young adult women caused by *Staphylococcus saprophyticus*. JAMA.

[10] Hovelius B., Mårdh P.A. (1984). *Staphylococcus saprophyticus* as a common cause of urinary tract infections. Rev. Infect. Dis..

[11] Schneider P.F., Riley T.V. (1996). *Staphylococcus saprophyticus* urinary tract infections: Epidemiological data from Western Australia. Eur. J. Epidemiol..

[12] Jordan P.A., Iravani A., Richard G.A., Baer H. (1980). Urinary tract infection caused by *Staphylococcus saprophyticus*. J. Infect. Dis..

[13] Garduño E., Márquez I., Beteta A., Said I., Blanco J., Pineda T. (2005). *Staphylococcus saprophyticus* causing native valve endocarditis. Scand. J. Infect. Dis..

[14] Tamura D., Yamane H., Tabakodani H., Yamagishi H., Nakazato E., Kimura Y., Shinjoh M., Yamagata T. (2021). Clinical impact of bacteremia due to *Staphylococcus saprophyticus*. Adv. Infect. Dis..

[15] Hur J., Lee A., Hong J., Jo W.Y., Cho O.H., Kim S., Bae I.G. (2016). *Staphylococcus saprophyticus* bacteremia originating from urinary tract infections: A case report and literature review. Infect. Chemother..

[16] Anderson J.D., Clarke A.M., Anderson M.E., Isaac-Renton J.L., McLoughlin M.G. (1981). Urinary tract infections due to *Staphylococcus saprophyticus* biotype 3. CMAJ.

[17] Golledge C.L. (1988). *Staphylococcus saprophyticus* bacteremia. J. Infect. Dis..

[18] Kline K.A., Kau A.L., Chen S.L., Lim A., Pinkner J.S., Rosch J., Nallapareddy S.R., Murray B.E., Henriques-Normark B., Beatty W. (2010). Characterization of a novel murine model of *Staphylococcus saprophyticus* urinary tract infection reveals roles for Ssp and SdrI in virulence. Infect. Immun..

[19] Hedman P., Ringertz O., Lindström M., Olsson K. (1993). The origin of *Staphylococcus saprophyticus* from cattle and pigs. Scand. J. Infect. Dis..

[20] Hedman P., Ringertz O., Eriksson B., Kvarnfors P., Andersson M., Bengtsson L., Olsson K. (1990). *Staphylococcus saprophyticus* found to be a common contaminant of food. J. Infect..

[21] Penna B., Varges R., Martins R., Martins G., Lilenbaum W. (2010). In vitro antimicrobial resistance of staphylococci isolated from canine urinary tract infection. Can. Vet. J..

[22] Hauschild T., Wójcik A. (2007). Species distribution and properties of staphylococci from canine dermatitis. Res. Vet. Sci..

[23] Mossakowski P., Lew-Kojrys S. (2023). Nietypowy rodzaj kamieni moczowych u psa—Węglan apatytu. Mag. Wet..

[24] Guo C., Sun W., Cheng W., Chen N., Lv Y. (2024). Isolation and characterization of *Staphylococcus saprophyticus* responsible for the death of two six-band-banded armadillos (*Euphractus sexcinctus*). Vet. Rec. Case Rep..

[25] Miszczak M., Korzeniowska-Kowal A., Wzorek A., Gamian A., Rypuła K., Bierowiec K. (2023). Colonization of methicillin-resistant *Staphylococcus* species in healthy and sick pets: Prevalence and risk factors. BMC Vet. Res..

[26] Kloos W.E., Bannerman T.L. (1994). Update on clinical significance of coagulase-negative staphylococci. Clin. Microbiol. Rev..

[27] Schulin T., Voss A. (2001). Coagulase-negative staphylococci as a cause of infections related to intravascular prosthetic devices: Limitations of present therapy. Clin. Microbiol. Infect..

[28] Giormezis N., Kolonitsiou F., Foka A., Drougka E., Liakopoulos A., Makri A., Papanastasiou A.D., Vogiatzi A., Dimitriou G., Marangos M. (2014). Coagulase-negative staphylococcal bloodstream and prosthetic-device-associated infections: The role of biofilm formation and distribution of adhesion and toxin genes. J. Med. Microbiol..

[29] May L., Klein E.Y., Rothman R.E., Laxminarayan R. (2014). Trends in antibiotic resistance in coagulase-negative staphylococci in the United States 1999 to 2012. Antimicrob. Agents Chemother..

[30] Marincola G., Liong O., Schoen C., Abouelfetouh A., Hamdy A., Wencker F.D.R., Marciniak T., Becker K., Köck R., Ziebuhr W. (2021). Antimicrobial resistance profiles of coagulase-negative staphylococci in community-based healthy individuals in Germany. Front. Public Health.

[31] Otto M. (2012). Coagulase-negative staphylococci as reservoirs of genes facilitating MRSA infection. Bioessays.

[32] Huang Y.S., Lai L.C., Chen Y.A., Lin K.Y., Chou Y.H., Chen H.C., Wang S.S., Wang J.T., Chang S.C. (2020). Colonization with multidrug-resistant organism among healthy adults in the community setting: Prevalence, risk factors, and composition of gut microbiome. Front. Microbiol..

[33] Diop M., Bassoum O., Ndong A., Wone F., Tamouh A.G., Ndoye M., Youbong T., Daffé S.M.M., Radji R.O., Gueye M.W. (2025). Prevalence of multidrug-resistant bacteria in healthcare and community settings in West Africa: Systematic review and meta-analysis. BMC Infect. Dis..

[34] Cristino J.A., Pereira A.T., Andrade L.G. (1989). Diversity of plasmids in *Staphylococcus saprophyticus* isolated from urinary tract infections in women. Epidemiol. Infect..

[35] Vickers A.A., Chopra I., O’Neill A.J. (2007). Intrinsic novobiocin resistance in *Staphylococcus saprophyticus*. Antimicrob. Agents Chemother..

[36] De Pavia-Santos W., Barros E.M., de Sousa V.S., Silva Laport M., Giambiagi-deMarval M. (2016). Identification of coagulase-negative *Staphylococcus saprophyticus* by PCR based on the heat-shock repressor encoding hrcA gene. Diagn. Microbiol. Infect. Dis..

[37] Clinical and Laboratory Standards Institute (CLSI) (2024). Method for Dilution Antimicrobial Susceptibility Test for Bacteria That Grow Aerobically.

[38] Clinical and Laboratory Standards Institute (CLSI) (2024). Performance Standards for Antimicrobial Disk and Dilution Suseptibility Test for Bacteria Isolated from Animals.

[39] Clinical and Laboratory Standards Institute (CLSI) (2024). Performance Standards for Antimicrobial Susceptibility Testing.

[40] European Committee on Antimicrobial Susceptibility Testing (EUCAST) (2024). Breakpoint Tables for Interpretation of MICs and Zone Diameters.

[41] Magiorakos A.P., Srinivasan A., Carey R.B., Carmeli Y., Falagas M.E., Giske C.G., Harbarth S., Hindler J.F., Kahlmeter G., Olsson Liljequist B. (2012). Multidrug-resistant, extensively drug-resistant and pandrug-resistant bacteria: An international expert proposal for interim standard definitions for acquired resistance. Clin. Microbiol. Infect..

[42] Marques C., Belas A., Franco A., Aboim C., Telo Gama L., Pomba C. (2022). Increase in antimicrobial resistance and emergence of major international high-risk clonal lineages in dogs and cats with urinary tract infection: 16-year retrospective study. BMC Vet. Res..

[43] Widerström M., Wiström J., Sjöstedt A., Monsen T. (2012). Coagulase-negative staphylococci: Update on the molecular epidemiology and clinical presentation, with a focus on *Staphylococcus epidermidis* and *Staphylococcus saprophyticus*. Eur. J. Clin. Microbiol. Infect. Dis..

[44] Cunha M.L.R.S., Sinzato Y.K., Silveira L.V.A. (2004). Comparison of methods for the identification of coagulase-negative staphylococci. Mem. Inst. Oswaldo Cruz.

[45] Kleeman K.T., Bannerman T.L. (1993). Evaluation of the Vitek System Gram-positive identification card for species identification of coagulase-negative staphylococci. J. Clin. Microbiol..

[46] Mlaga K.D., Dubourg G., Abat C., Chaudet H., Lotte L., Diene S.M., Raoult D., Ruimy R., Rolain J.M. (2017). Using MALDI-TOF MS typing method to decipher outbreak: The case of *Staphylococcus saprophyticus* causing urinary tract infections (UTIs) in Marseille, France. Eur. J. Clin. Microbiol. Infect. Dis..

[47] Dubois D., Leyssene D., Chacornac J.P., Kostrzewa M., Schmit P.O., Talon R., Bonnet R., Delmas J. (2010). Identification of a variety of *Staphylococcus* species by matrix-assisted laser desorption ionization–time of flight mass spectrometry. J. Clin. Microbiol..

[48] Zhang K., Potter R.F., Marino J., Muenks C.E., Lammers M.G., Dien Bard J., Dingle T.C., Humphries R., Westblade L.F., Burnham C.A.D. (2023). Comparative genomics reveals the correlations of stress response genes and bacteriophages in developing antibiotic resistance of *Staphylococcus saprophyticus*. mSystems.

[49] Youngblom M.A., Imhoff M.R., Smyth L.M., Mohamed M.A., Pepperell C.S. (2023). Portrait of a generalist bacterium: Pathoadaptation, metabolic specialization and extreme environments shape diversity of *Staphylococcus saprophyticus*. BioRxiv.

[50] Lawal O.U., Fraqueza M.J., Bouchami O., Worning P., Bartels M.D., Gonçalves M.L., Paixão P., Gonçalves E., Toscano C., Empel J. (2021). Foodborne origin and local and global spread of *Staphylococcus saprophyticus* causing human urinary tract infections. Emerg. Infect. Dis..

[51] Sousa V.S., Rabello R.F., Dias R.C.S., Martins I.S., Santos L.B.G.S., Alves E.M., Riley L.W., Moreira B.M. (2013). Time-based distribution of *Staphylococcus saprophyticus* pulsed-field gel-electrophoresis clusters in community-acquired urinary tract infections. Mem. Inst. Oswaldo Cruz.

[52] Rafiee M., Tabarraei A., Yazdi M., Mohebbi A., Ghaemi E.A. (2023). Antimicrobial resistance patterns of *Staphylococcus saprophyticus* isolates causing urinary tract infections in Gorgan, North of Iran. Med. Lab. J..

[53] Khan F., Haadi S., Khan F.A., Shakir J., Shafiq M., Tariq S., Ahmad J., Afzal Q., Khan A.A., Afridi P. (2023). Antibiotic susceptibility profile of *Staphylococcus saprophyticus* isolated from clinical samples in Peshawar, Pakistan. Sciencetech.

[54] Marepalli N.R., Nadipelli A.R., Jain M.K., Parnam L.S., Vashyani A. (2024). Patterns of Antibiotic Resistance in Urinary Tract Infections: A Retrospective Observational Study. Cureus.

[55] Ferreira A.M., Bonesso M.F., Mondelli A.L., Camargo C.H., Cunha M.L.R.S. (2002). Oxacillin resistance and antimicrobial susceptibility profile of *Staphylococcus saprophyticus*. Chemotherapy.

[56] Chambers H.F. (2001). The changing epidemiology of *Staphylococcus aureus*?. Emerg. Infect. Dis..

[57] Lee B., Jeong D.W., Lee J.H. (2015). Genetic diversity and antibiotic resistance of *Staphylococcus saprophyticus* isolates from fermented foods and clinical samples. J. Korean Soc. Appl. Biol. Chem..

[58] Chua K.Y.L., Yang M., Wong L., Knox J., Lee L.Y. (2023). Antimicrobial resistance and its detection in *Staphylococcus saprophyticus* urinary isolates. Pathology.

[59] Schmitz F.J., Theis S., Fluit A.C., Verhoef J., Heinz H.P., Jones M.E. (1999). Antimicrobial susceptibility of coagulase-negative staphylococci isolated between 1991 and 1996 from a German university hospital. Clin. Microbiol. Infect..

[60] Yang Y., Li Y., Wang X., Zhang H., Liu J. (2023). Antimicrobial resistance and virulence profiles of staphylococci from clinical bovine mastitis in Ningxia Hui Autonomous Region of China. Front. Microbiol..

[61] Amiri R., Alipour M., Engasi A.K., Amiri A.R., Mofarrah R. (2023). Monitoring and investigation of resistance genes *gyrA, parC, blaZ, ermA, ermB* and ermC in *Staphylococcus saprophyticus* isolated from urinary tract infections in Mazandaran Province, Iran. Infect. Epidemiol. Microbiol..

[62] Yang Y., Hu X., Cai S., Hu N., Yuan Y., Wu Y., Wang Y., Mi J., Liao X. (2023). Pet cats may shape the antibiotic resistome of their owner’s gut and living environment. Microbiome.

[63] Guardabassi L., Schwarz S., Lloyd D.H. (2004). Pet animals are reservoirs of antimicrobial resistant bacteria: Review. J. Antimicrob. Chemother..

[64] Yazdankhah S.P., Asli A.W., Sorum H., Oppegaard H., Sunde M. (2006). Fusidic acid resistance, mediated by *fusB* in bovine coagulase-negative staphylococci. J. Antimicrob. Chemother..

[65] Patel J.B., Gorwitz R.J., Jernigan J.A. (2009). Mupirocin resistance. Clin. Infect. Dis..

[66] Lloyd D.H. (2007). Reservoirs of antimicrobial resistance in pet animals. Clin. Infect. Dis..

[67] Hiramatsu K., Katayama Y., Matsuo M., Sasaki T., Morimoto Y., Sekiguchi A., Baba T. (2014). Multi-drug-resistant *Staphylococcus aureus* and future chemotherapy. J. Infect. Chemother..

[68] Gupta K., Hooton T.M., Naber K.G., Wullt B., Colgan R., Miller L.G., Moran G.J., Nicolle L.E., Raz R., Schaeffer A.J. (2011). Updated recommendations: International clinical practice guidelines for the treatment of acute uncomplicated cystitis and pyelonephritis in women. Clin. Infect. Dis..

[69] Stevens D.L., Bisno A.L., Chambers H.F., Dellinger E.P., Goldstein E.J.C., Gorbach S.L., Hirschmann J.V., Kaplan S.L., Montoya J.G., Wade J.C. (2014). Practice guidelines for the diagnosis and management of skin and soft tissue infections: 2014 update by the Infectious Diseases Society of America. Clin. Infect. Dis..

[70] Weese J.S., Blondeau J., Boothe D., Breitschwerdt E., Guardabassi L., Hillier A., Lloyd D.H., Papich M.G., Rankin S., Turnidge J. (2019). ISCAID guidelines for the diagnosis and management of bacterial urinary tract infections in dogs and cats. J. Vet. Intern. Med..

[71] Hillier A., Lloyd D.H., Weese J.S., Blondeau J.M., Boothe D., Breitschwerdt E., Guardabassi L., Papich M.G., Rankin S., Turnidge J.D. (2014). Guidelines for the diagnosis and antimicrobial therapy of canine superficial bacterial folliculitis (Antimicrobial Guidelines Working Group of the International Society for Companion Animal Infectious Diseases). Vet. Dermatol..

[72] Archer N.K., Mazaitis M.J., Costerton J.W., Leid J.G., Powers M.E., Shirtliff M.E. (2011). *Staphylococcus aureus* biofilms: Properties, regulation, and roles in human disease. Virulence.

[73] Tsai C.J.Y., Loh J.M.S., Proft T. (2016). *Galleria mellonella* infection models for the study of bacterial diseases and for antimicrobial drug testing. Virulence.

[74] Koksal F., Yasar H., Samasti M. (2009). Antibiotic resistance patterns of coagulase-negative *Staphylococcus* strains isolated from blood cultures of septicemic patients in Turkey. Microbiol. Res..

[75] Desbois A.P., Coote P.J. (2011). Wax moth larva (*Galleria mellonella*): An in vivo model for assessing the efficacy of antistaphylococcal agents. J. Antimicrob. Chemother..

[76] Champion O.L., Wagley S., Titball R.W. (2016). *Galleria mellonella* as a model host for microbiological and toxin research. Virulence.

[77] Bierowiec K., Korzeniowska-Kowal A., Wzorek A., Rypuła K., Gamian A. (2019). Prevalence of *Staphylococcus* species colonization in healthy and sick cats. Biomed. Res. Int..

[78] Samad R.A., Al Disi Z., Ashfaq M.Y.M., Wahib S.M., Zouari N. (2020). The use of principle component analysis and MALDI-TOF MS for the differentiation of mineral forming *Virgibacillus* and *Bacillus* species isolated from sabkhas. RSC Adv..

[79] Amini R., As A., Chung C., Jahanshiri F., Wong C.B., Poyling B., Hematian A., Sekawi Z., Zargar M., Jalilian F.A. (2012). Circulation and transmission of methicillin-resistant *Staphylococcus aureus* among college students in Malaysia (cell phones as reservoir). Asian Biomed..

[80] Ullah F., Malik S.A., Ahmed J., Ullah F., Shah S.M., Ayaz M., Hussain S., Khatoon L. (2012). Investigation of the genetic basis of tetracycline resistance in *Staphylococcus aureus* from Pakistan. Trop. J. Pharm. Res..

[81] Saadat S., Solhjoo K., Norooz-Nejad M.J., Kazemi A. (2014). *VanA* and *VanB* positive vancomycin resistant *Staphylococcus aureus* among clinical isolates in Shiraz, South Iran. Oman Med. J..

[82] Ciesielczuk H., Xenophontos M., Lambourne J. (2019). Methicillin-resistant *Staphylococcus aureus* harboring *mecC* still eludes us in East London, United Kingdom. J. Clin. Microbiol..

[83] O’Neill A., Chopra I. (2006). Molecular basis of *fusB*-mediated resistance to fusidic acid in *Staphylococcus aureus*. Mol. Microbiol..

[84] Tasara T., Cernela N., Stephan R. (2013). Function impairing mutations in *blaZ* and *blaR* genes of penicillin susceptible *Staphylococcus aureus* strains isolated from bovine mastitis. Schweiz. Arch. Tierheilkd..

[85] Šeol B. (2005). Comparative in vitro activities of enrofloxacin, ciprofloxacin and marbofloxacin against *Staphylococcus intermedius* isolated from dogs. Vet. Arh..

[86] Płoneczka-Janeczko K., Bierowiec K., Lis P., Rypuła K. (2014). Identification of bap and *icaA* genes involved in biofilm formation in coagulase-negative staphylococci isolated from feline conjunctiva. Vet. Res. Commun..

