# Supplementary material for: Characteristics of Staphylococcus saprophyticus Isolated from Humans and Animals
Source: Int J Mol Sci. 2025 Jul 17;26(14):6885. doi: 10.3390/ijms26146885 (PMC12295385; doi:10.3390/ijms26146885)
Supplement: Supplementary file 1 [file ijms-26-06885-s001.zip › Supplementary Figures.pdf]

Supplementary Materials

Characteristics of *Staphylococcus saprophyticus* Isolated from Humans and Animals

Paulina Prorok [1,*], Karolina Bierowiec [1,*], Milena Skrok [1], Magdalena Karwańska [1], Magdalena Siedlecka [1], Marta Miszczak [1], Marta Książczyk [2], Katarzyna Kapczyńska [3] and Krzysztof Rypuła [1]

[1] Department of Epizootiology and Clinic of Birds and Exotic Animals, Division of Infectious Diseases and Veterinary Sciences, 50-365 Wrocław, Poland; milena.skrok@upwr.edu.pl (M.S.); magdalena.karwanska@upwr.edu.pl (M.K.); magdalena.siedlecka@upwr.edu.pl (M.S.); marta.miszczak@upwr.edu.pl (M.M.); krzysztof.rypula@upwr.edu.pl (K.R.)

[2] Department of Microbiology, Faculty of Biological Sciences, University of Wroclaw, 51-148 Wrocław, Poland; marta.ksiazczyk@uwr.edu.pl

[3] Laboratory of Medical Microbiology, Hirszfeld Institute of Immunology and Experimental Therapy, Polish Academy of Sciences, 53-114 Wrocław, Poland; katarzyna.kapczynska@hirszfeld.pl

* Correspondence: paulina.prorok@upwr.edu.pl (P.P.); karolina.bierowiec@upwr.edu.pl (K.B.)

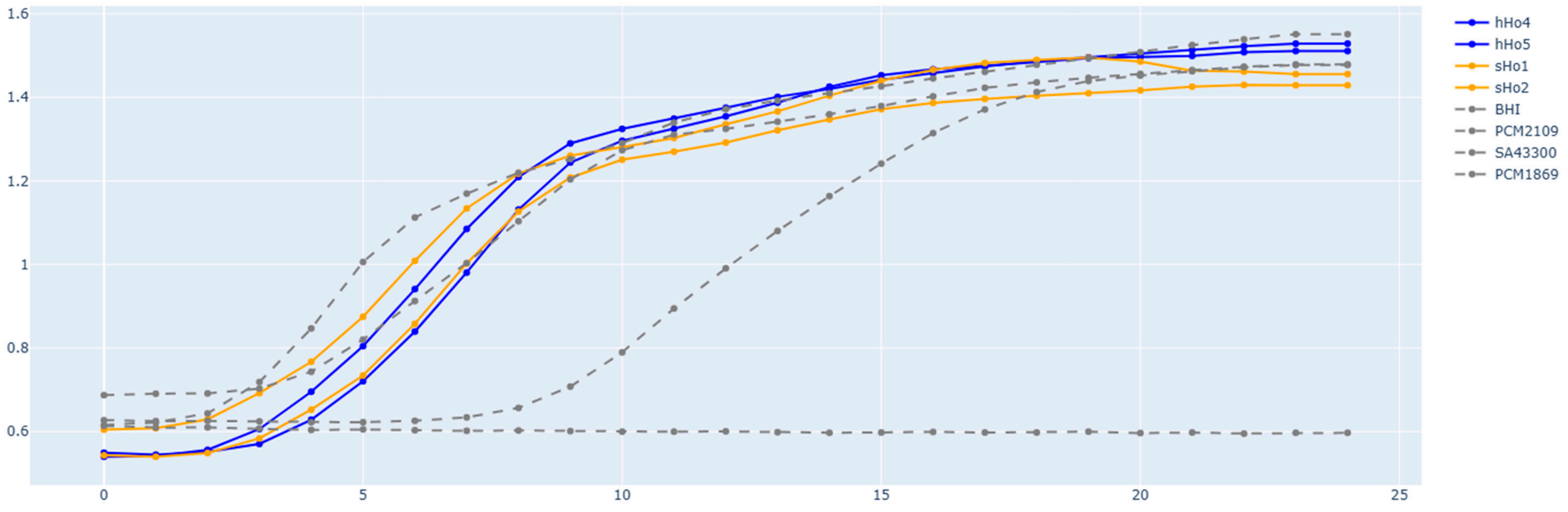

Figure S1. Growth curves (Human) 37°C.

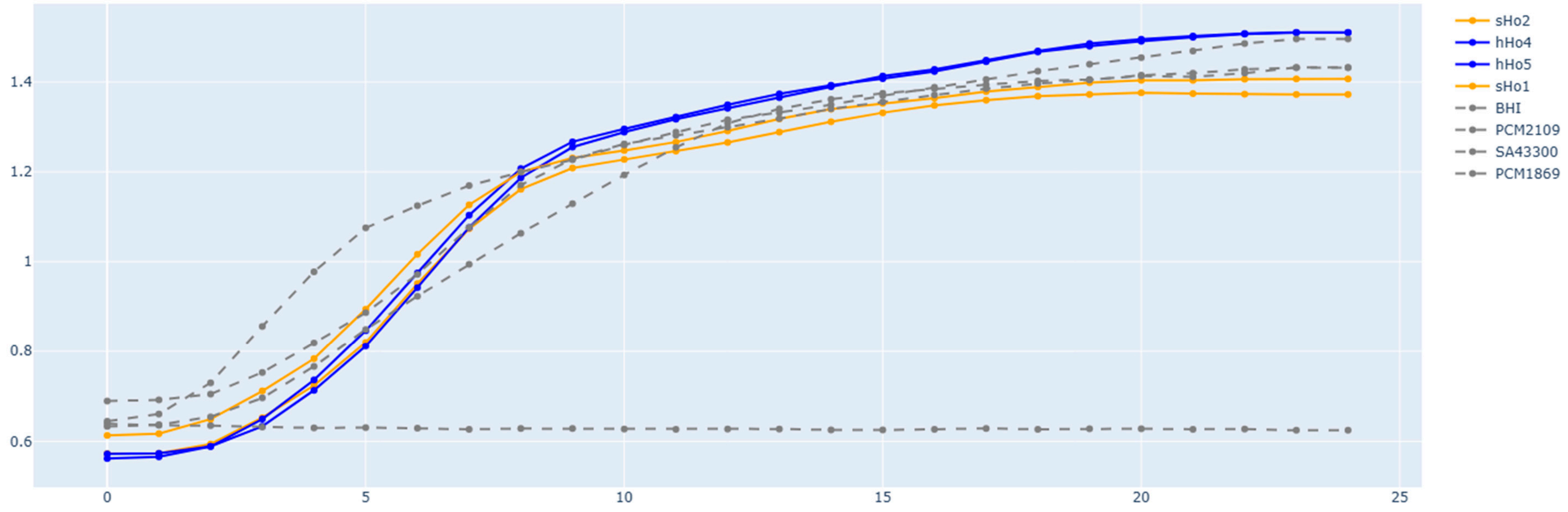

Figure S2. Growth curves (Human) 38°C.

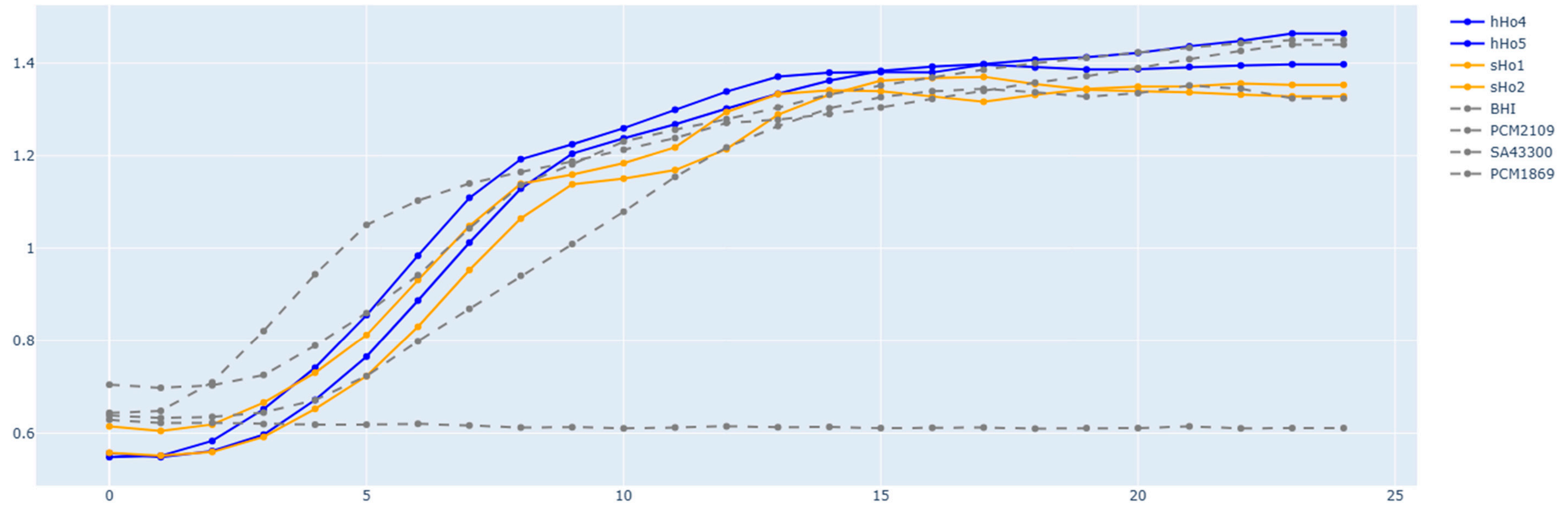

Figure S3. Growth curves (Human) 39°C.

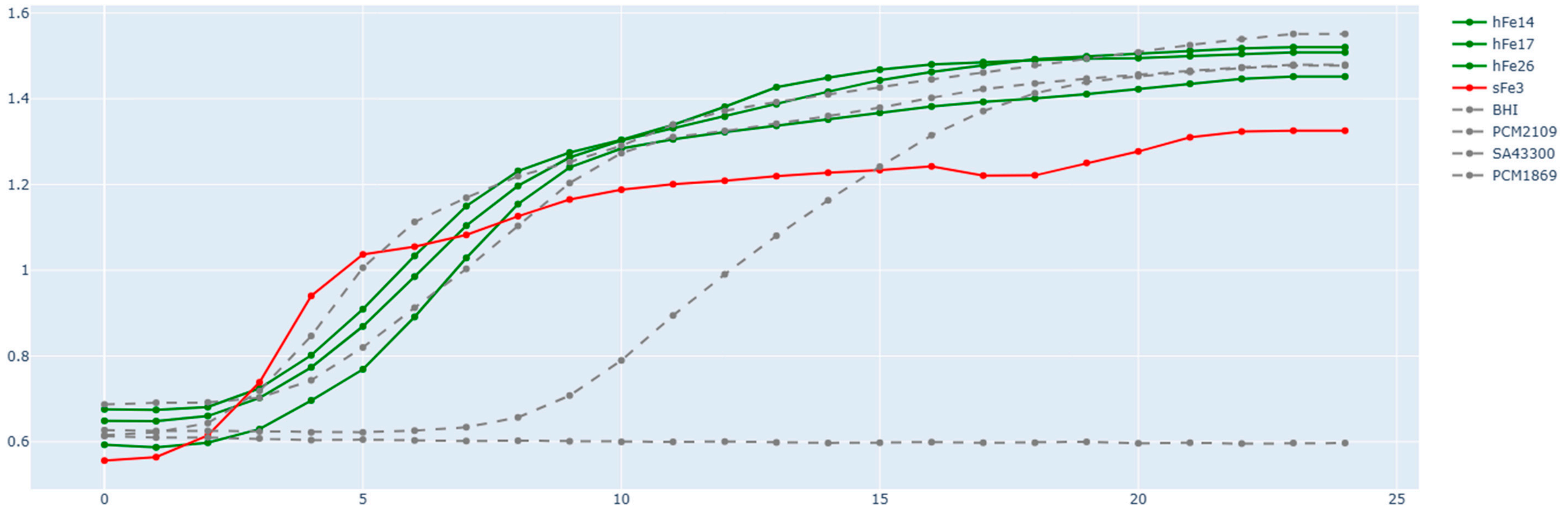

Figure S4. Growth curves (Feline) 37°C.

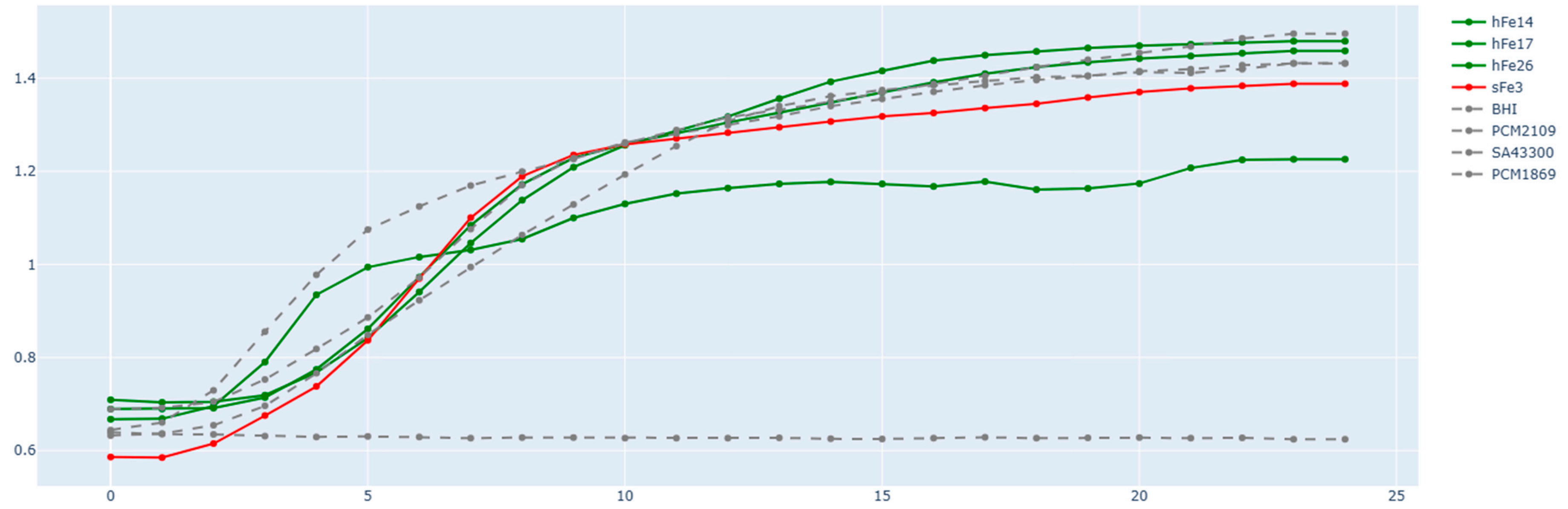

Figure S5. Growth curves (Feline) 38°C.

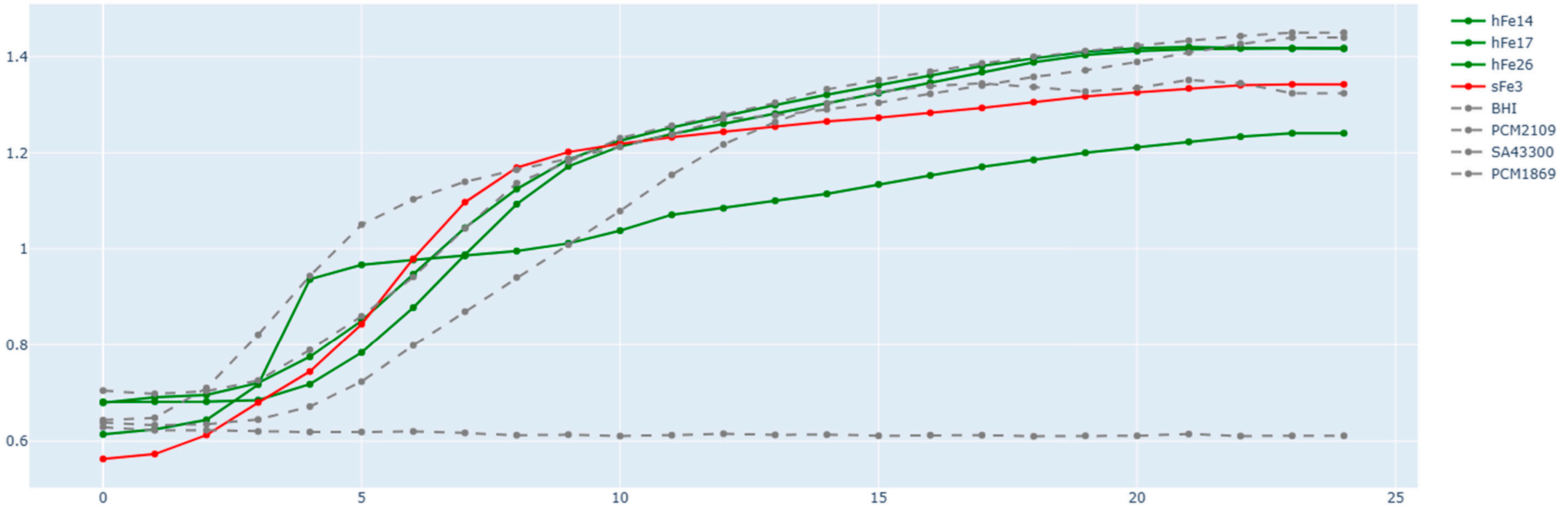

Figure S6. Growth curves (Feline) 39°C.

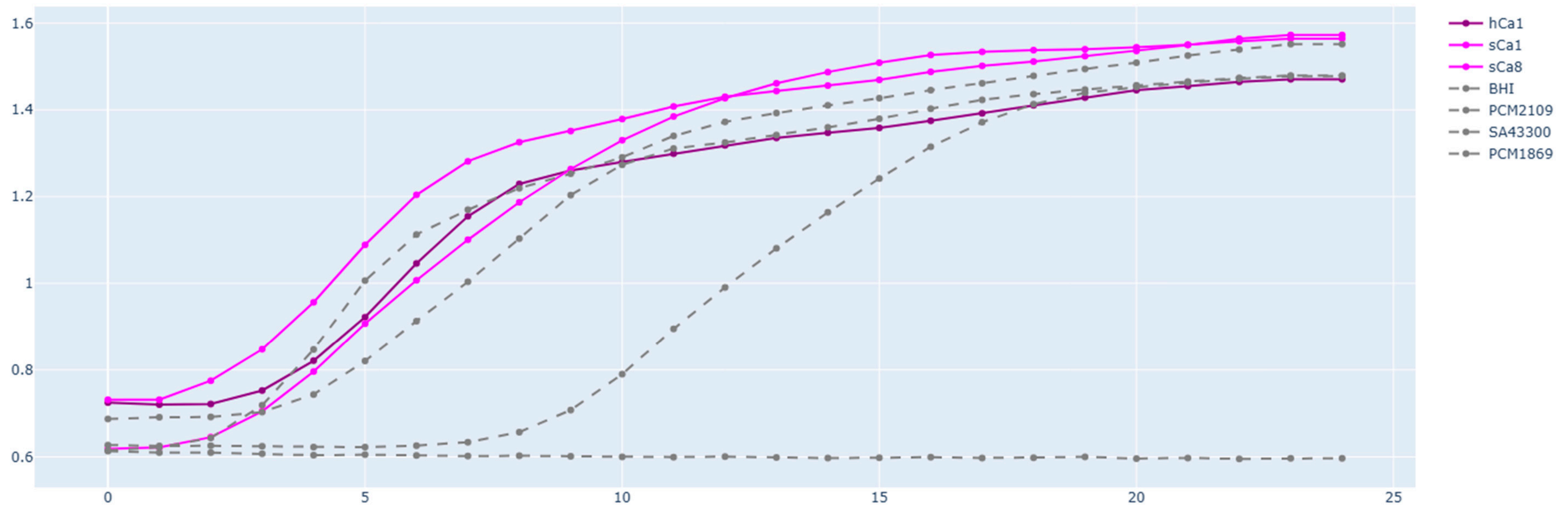

Figure S7. Growth curves (Canine) 37°C.

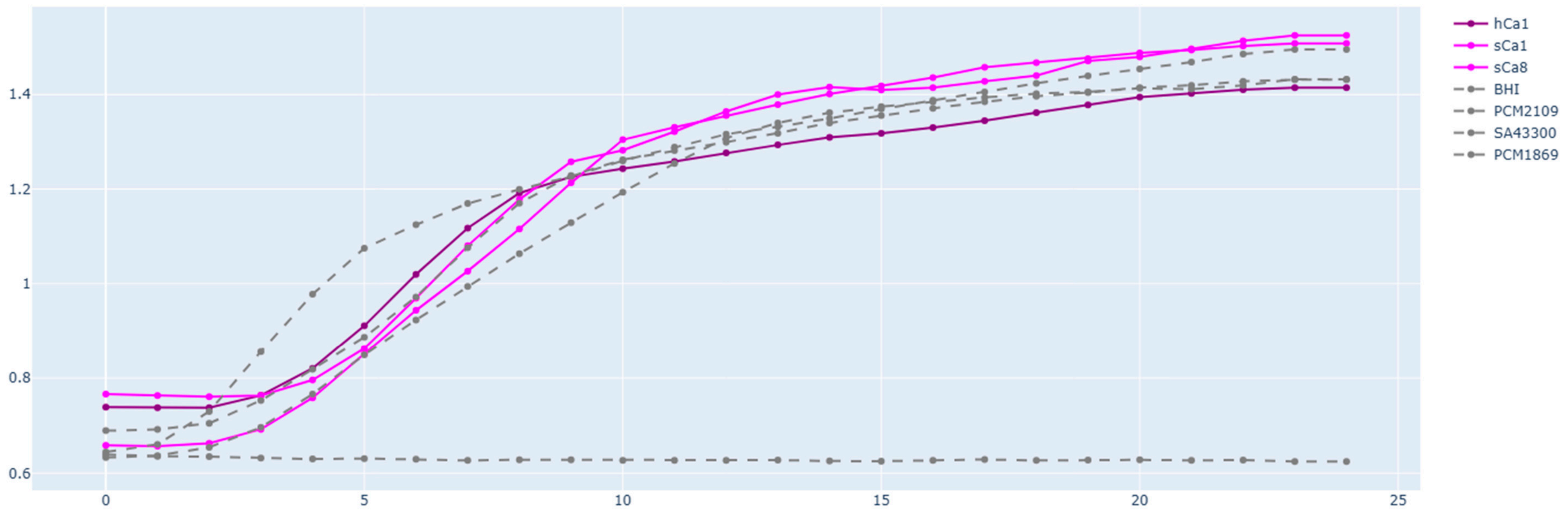

Figure S8. Growth curves (Canine) 38°C.

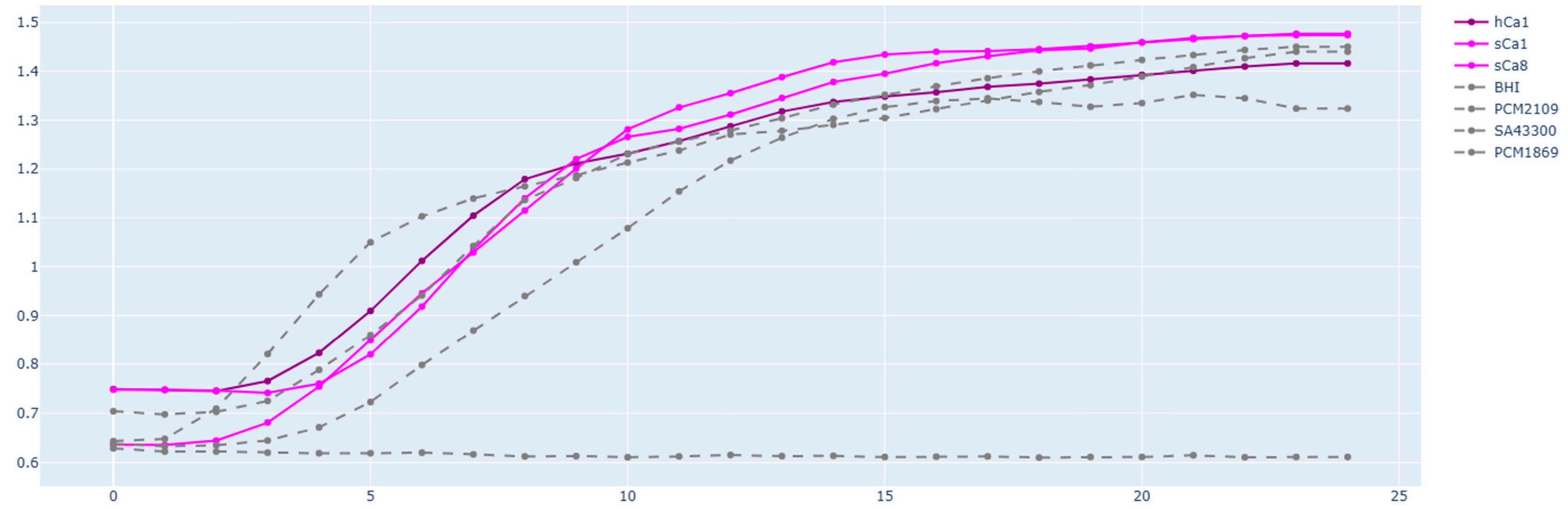

Figure S9. Growth curves (Canine) 39°C.

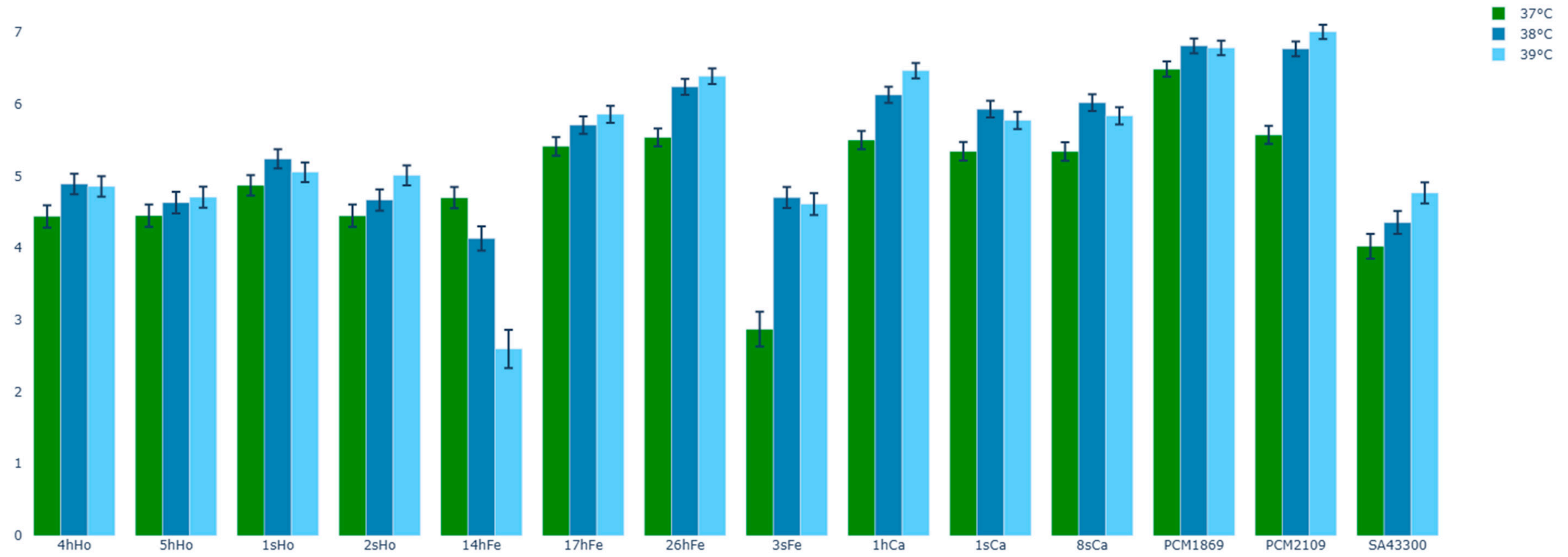

Figure S10. Doubling time by strain and temperature.
